# Superprotonic Conduction in Donor Co‐Doped Perovskites

**DOI:** 10.1002/anie.202521773

**Published:** 2026-01-19

**Authors:** Kensei Umeda, Kei Saito, Takashi Honda, Masatomo Yashima

**Affiliations:** ^1^ Department of Chemistry School of Science Institute of Science Tokyo 2‐12‐1‐W4‐17, O‐okayama Meguro‐ku Tokyo 152‐8551 Japan; ^2^ Institute of Materials Structure Science High Energy Accelerator Research Organization (KEK) Tsukuba Ibaraki 305‐0801 Japan; ^3^ J‐PARC Center High Energy Accelerator Research Organization (KEK) Tokai Ibaraki 319‐1106 Japan; ^4^ Center for Energy Systems Design (CESD) International Institute for Carbon Neutral Energy Research (WPI‐I^2^CNER) Kyushu University 744 Motooka Nishi‐ku Fukuoka 819‐0395 Japan

**Keywords:** Materials science, Neutron diffraction, Novel materials, Perovskite phase, Proton conductors

## Abstract

Donor doping of oxygen‐deficient BaScO_2.5_ is an unexplored strategy for achieving high proton conductivity at intermediate temperatures of 200−400 °C. In this work, a new series of BaSc_1−_ *_x_*_−_ *_y_* Mo*_x_* W*_y_* O_3−_ *_δ_* compounds was prepared via Mo/W donor co‐doping where *x* is the Mo content, *y* is the W content, and *δ* is the amount of oxygen vacancies. The present work reports the enhancement of proton conductivity by the Mo/W donor co‐doping of BaScO_2.5_. BaSc_0.8_Mo_0.1_W_0.1_O_2.8_ exhibits exceptional proton conductivity—0.10 S cm^−^^1^ at 315 °C and 0.01 S cm^−^^1^ at 193 °C—alongside outstanding chemical stability in CO_2_, O_2_, and H_2_ atmospheres. The high proton conductivity originates from the synergistic effects of abundant oxygen vacancies (*δ* = 0.2) and full hydration, yielding a high proton concentration, coupled with high proton diffusivity. The high diffusivity is attributable to the reduced proton trapping compared with acceptor and isovalent doping and overdoping due to higher proton concentration. In contrast to the acceptor co‐doping, the donor co‐doping does not increase the activation energy, resulting in lower activation energy and higher proton conductivity. These findings establish donor co‐doping into the oxygen‐deficient perovskites as a powerful design principle for next‐generation proton conductors with high proton conductivity at intermediate temperatures.

## Graphical abstract

Ceramic proton conductors are promising materials for various applications such as fuel cells. Here, we report superprotonic conduction of BaSc_0.8_Mo_0.1_W_0.1_O_2.8_ created by donor Mo/W co‐doping into BaScO_2.5_ (e.g., 10^−2^ S cm^−1^ at 193 °C). The high proton conductivity is attributed to high carrier concentration and mobility. The present findings would open the new windows in the science and technology of donor co‐doped proton conductors.

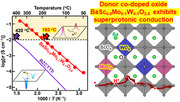

## Introduction

Hydrogen economy heavily relies on the efficient energy conversion between hydrogen and electricity.^[^ ^1^, ^2^, ^3^, ^4^, ^5^, ^6^, ^7^ ^]^ Solid oxide cells appear to be a promising solution. They can operate as switchable electrochemical devices, functioning as either fuel cells (converting hydrogen to electricity) or electrolysis cells (converting electricity to hydrogen).^[^ ^1^ **^,^** ^8^, ^9^, ^10^, ^11^, ^12^, ^13^ ^]^ Proton conductors generally exhibit higher ionic conductivity compared with oxide‐ion conductors at intermediate temperatures (200−400 °C). This has led to greater interests in protonic ceramic fuel cells (PCFCs) and electrolysis cells (PCECs) compared with conventional solid oxide fuel cells (SOFCs) and electrolysis cells (SOECs).^[^ ^14^, ^15^, ^16^ ^]^ High proton conductors with high chemical stability are needed to develop high‐performance PCFCs and PCECs.^[^ ^17^ ^]^

In general, polymers such as Nafion and salts exhibit high proton conductivity at low temperatures (50−200 °C); however, their chemical stability deteriorates significantly at intermediate temperatures.^[^ ^18^, ^19^, ^20^, ^21^ ^]^ In contrast, oxides offer excellent chemical stability but have low proton conductivity in the same temperature range. Consequently, there are no known ionic conductors that combine high ionic conductivity with high chemical stability at intermediate temperatures. Norby referred to the region of conductivity and temperature lacking suitable materials as the “gap.” The absence of viable candidates in this “Norby gap” has driven the search for new ionic conductors.^[^ ^22^ **^,^** ^23^ ^]^

Since Iwahara's pioneering discovery of proton conduction in acceptor‐doped SrCeO_3_ in 1981,^[^ ^24^ ^]^ extensive research has focused on the acceptor‐doped perovskite‐type proton conductors, such as *AB* ^4+^_1−_ *_x_M* ^3+^ *_x_* O_3−_ *_δ_*, in which the trivalent acceptor cations (*M* ^3+^) are partially substituted for the host cations *B* ^4+^ in the parent *AB* ^4+^O_3_ perovskites without oxygen vacancies.^[^ ^22^ **^,^** ^25^, ^26^, ^27^, ^28^, ^29^, ^30^, ^31^ ^]^ In these materials, *A* is a relatively large *A*‐site cation, while the *B* and *M* are relatively small *B*‐site cations, the *x* is the dopant *M* content, and the *δ* is the amount of oxygen vacancies. The acceptor *M* ^3+^ is a dopant cation with a lower oxidation number than the host cation *B* ^4+^. A well‐known example is the Y‐doped BaZrO_3_ (*A* = Ba, *B* = Zr, *M* = Y), widely regarded as a benchmark proton conductor.^[^ ^32^, ^33^, ^34^ ^]^

**Figure 1:**
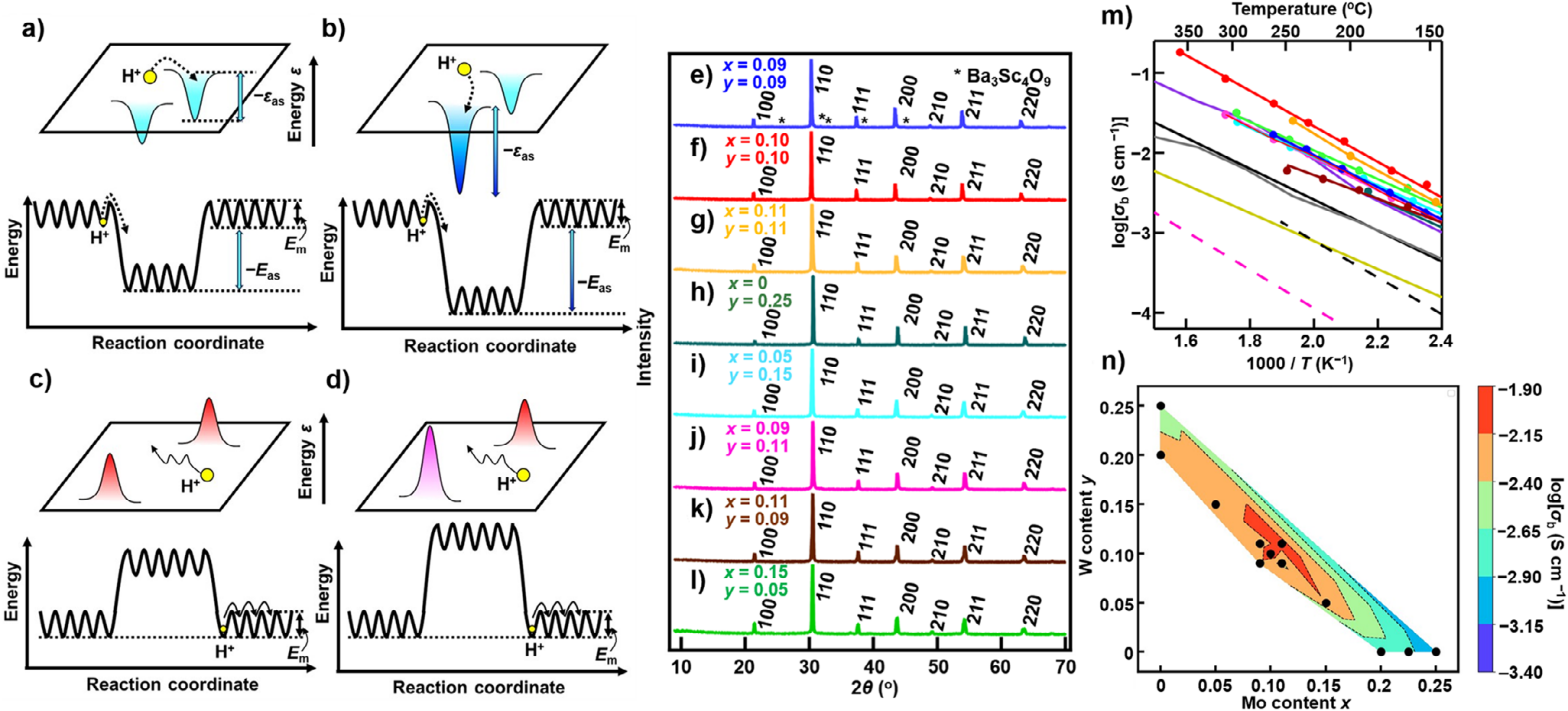
High bulk conductivity of Mo/W co‐doped BaScO_2.5_ materials, BaSc_1−_ *_x_*_−_ *_y_* Mo*_x_* W*_y_* O_3−_ *_δ_* in wet air, showing the highest conductivity of BSM10W10. Schematic energy diagrams of a proton in a) an acceptor mono‐doped material, b) an acceptor co‐doped material, c) a donor mono‐doped material, and d) a donor co‐doped material. The upper part of each panel illustrates the local energy landscape of a proton *ε*, while the lower part in each panel represents the macroscopic energy diagram of a proton. The yellow circle denotes a proton. In panels (a) and (b), protons can be trapped at the light blue and blue stable positions near oxide ions coordinated with different acceptor dopants. In panels (c) and (d), protons cannot be trapped at the red and violet unstable positions near oxide ions coordinated with different donor dopants. *E*_m_ is the intrinsic (trap‐free) energy barrier for proton migration without association. *E*_as_ denotes the macroscopic association energy between a proton and an acceptor dopant. The acceptor co‐doping increases |*E*_as_| (lower part of panel (b)), due to increased structural disorder leading to the formation of a local deeper trap site with a larger trap energy |*ε*_as_| (upper part of panel (b)). In contrast, the donor co‐doping does not increase significantly |*E*_as_| due to lesser influence of increased disorder around the donor dopant on the ScO_6_ octahedral network. Therefore, the activation energy *E*_a_ = *E*_m_ + |*E*_as_| of donor co‐doped materials is lower than that of acceptor co‐doped ones. Cu Kα X‐ray powder diffraction patterns of as‐prepared e) BSM9W9, f) BSM10W10, g) BSM11W11, h) BSW25, i) BSM5W15, j) BSM9W11, k) BSM11W9, and l) BSM15W5 powders at 24 °C. *hkl* denotes the reflection index of the primitive cubic cell. Asterisk marks denote the impurity phase of Ba_3_Sc_4_O_9_. m) Arrhenius plots of the bulk conductivity *σ*_b_ of BSM9W9 (blue circles and line), BSM10W10(red circles and line), BSM11W11 (orange circles and line), BSW25 (dark green circle and line), BSW20 (purple line),^[^ ^40^ ^]^ BSM5W15 (light blue circles and line), BSM9W11 (pink circles and line), BSM11W9 (brown circles and line), BSM15W5 (light green circles and line), BSM20 (black line),^[^ ^23^ ^]^ BSM22.5 (gray line),^[^ ^43^ ^]^ and BSM25 (yellow line)^[^ ^23^ ^]^ in wet air. See the Table S1 for the chemical compositions. Pink and black dashed lines represent BaCe_0.9_Y_0.1_O_2.95_ (BCY)^[^ ^25^ ^]^ and BaZr_0.8_Y_0.2_O_2.9_ (BZY),^[^ ^32^ ^]^ respectively. n) Contours of the *σ*_b_ for the system BaSc_1−_ *_x_*_−_ *_y_* Mo*_x_* W*_y_* O_3−_ *_δ_* at 200 °C. The dashed line in the panel (n) was calculated assuming linear variation between data points.

Recently, several research groups have studied proton conduction in the oxides created by acceptor co‐doping, including high‐entropy oxides.^[^ ^17^, ^35^, ^36^, ^37^ ^]^ For instance, Choi et al. reported high chemical stability and notable proton conductivity in BaZr_0.4_Ce_0.4_Y_0.1_Yb_0.1_O_2.9_ created by Y/Yb acceptor co‐doping.^[^ ^14^ ^]^ Skubida et al. demonstrated proton conduction in high‐entropy oxide BaZr_1/8_Hf_1/8_Sn_1/8_Ti_1/8_Y_1/8_In_1/8_Sm_1/8_Yb_1/8_O_2.75_ created by Y/In/Sm/Yb acceptor co‐doping.^[^ ^38^ ^]^ However, the proton conductivity of these acceptor co‐doped materials remains moderate. One of the key challenges in such proton conductors is proton trapping.^[^ ^39^ ^]^ This is caused by the association of a proton defect, ${\left(\mathrm{OH}\right)}_{O}^{•}$ (or proton H^+^) with an acceptor dopant ($M_{B}^{\prime }$) having an effective negative charge of −1 due to the electrostatic attraction. The defect reaction can be expressed using the Kröger–Vink notation as:
(1)

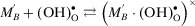

where ${\left(M_{B}^{\prime }\cdot {\left(\mathrm{OH}\right)}_{O}^{•}\right)}^{\times }$ is the proton–dopant association (Figure 1a). Proton trapping increases apparent activation energy for proton diffusivity and markedly reduces the conductivity at intermediate temperatures. In acceptor co‐doped oxides, two or more acceptor dopant elements can enhance proton trapping (Figure 1b), leading to pronounced decrease in conductivity. Consequently, achieving high proton conductivity at intermediate temperatures remains a great challenge for the acceptor co‐doped oxides.

In sharp contrast to the acceptor doping, donor *M* ^6+^ doping into hypothetical BaSc^3+^O_2.5_ ( = BaSc^3+^O_2.5_v_0.5_) with “intrinsic oxygen vacancies” v is an effective strategy to reduce the proton trapping (*M* ^6+^: Mo^6+^ and W^6+^) and stabilize the cubic perovskite phase.^[^ ^23^ ^]^ This enables high proton conductivity at intermediate temperatures.^[^ ^23^, ^40^, ^41^ ^]^ In this case, the donor dopant cation *M* ^6+^ has higher oxidation number than the host cation Sc^3+^, and the “intrinsic oxygen vacancies” refer to the structural oxygen vacancies in a parent material. The donor dopant cation *M* ^6+^ has a positive effective charge of + 3 relative to the host cation Sc^3+^. This prevents the proton–dopant association $(M_{B}^{•••}\cdot {{\left(\mathrm{OH}\right)}_{O}^{•})}^{••••}$ and the proton trapping via the donor dopant *M* ^6+^, because the *M* ^6+^ does not attract positively charged protons H^+^ (Figure 1c).

(2) $$M_{B}^{•••}+{\left(\mathrm{OH}\right)}_{O}^{•}⇄{\left(M_{B}^{•••}\cdot {\left(\mathrm{OH}\right)}_{O}^{•}\right)}^{••••}$$

For instance, Saito and Yashima reported that doping with the donor element molybdenum (Mo^6+^) in BaSc^3+^O_2.5_ stabilizes the cubic perovskite phase BaSc_0.8_Mo_0.2_O_2.8_, which exhibits high chemical stability and remarkable proton conductivity within the “Norby gap” (e.g., 10 mS cm^−1^ at 320 °C).^[^ ^23^ ^]^ Subsequently, Saito et al. have reported even higher proton conductivity of BaSc_0.8_W_0.2_O_2.8_ (e.g., 10 mS cm^−1^ at 235 °C).^[^ ^40^ ^]^ Compared to the acceptor co‐doped oxides, the donor co‐doped materials are expected to exhibit remarkably high proton conductivity at intermediate temperatures due to the reduced proton trapping (Figure 1d). However, to the best of our knowledge, proton conductors created by donor co‐doping have not yet been reported.

This study reports remarkably high proton conductivity in novel materials created by Mo/W donor co‐doping into the parent material BaScO_2.5_, with intrinsic oxygen vacancies (BaSc_1−_ *_x_*_−_ *_y_* Mo*_x_* W*_y_* O_2.5+3_ *_x_*_/2+3_ *_y_*_/2_; *x*: Mo content, *y*: W content). In particular, it will be shown that BaSc_0.8_Mo_0.1_W_0.1_O_2.8_ exhibits the highest proton conductivity among ceramic proton conductors (e.g., 0.1 S cm^−1^ at 315 °C and 0.01 S cm^−1^ at 193 °C). We selected the chemical compositions BaSc_1−_ *_x_*_−_ *_y_* Mo*_x_* W*_y_* O_2.5+3_ *_x_*_/2+3_ *_y_*_/2_, because i) Both Mo^6+^‐ and W^6+^‐donor mono‐doped BaScO_2.5_ materials such as BaSc_0.8_Mo_0.2_O_2.8_ and BaSc_0.8_W_0.2_O_2.8_ exhibit high proton conductivity^[^ ^23^, ^40^ ^]^ and ii) Mo‐ and W‐containing oxides such as La_28_(W_0.7_Mo_0.3_)_4_O_54_ show significant proton conductivity.^[^ ^42^ ^]^

## Results and Discussion

New materials, BaSc_1−_ *_x_*_−_ *_y_* Mo*_x_* W*_y_* O_2.5+3_ *_x_*_/2+3_ *_y_*_/2−_ *_z_*_/2_(OH)*_z_* ( = BaSc_1−_ *_x_*_−_ *_y_* Mo*_x_* W*_y_* O_2.5+3_ *_x_*_/2+3_ *_y_*_/2+_ *_z_*_/2 _H*_z_ *= BaSc_1−_ *_x_*_−_ *_y_* Mo*_x_* W*_y_* O_2.5+3_ *_x_*_/2+3_ *_y_*_/2_(H_2_O)*_z_*_/2_), were synthesized by solid‐state reactions, where *z* is the proton concentration. The eight chemical compositions investigated were BaSc_0.82_Mo_0.09_W_0.09_O_3−_ *_δ_* (BSM9W9), BaSc_0.8_Mo_0.1_W_0.1_O_3−_ *_δ_* (BSM10W10), BaSc_0.78_Mo_0.11_W_0.11_O_3−_ *_δ_* (BSM11W11), BaSc_0.75_W_0.25_O_3−_ *_δ_* (BSW25), BaSc_0.8_Mo_0.05_W_0.15_O_3−_ *_δ_* (BSM5W15), BaSc_0.8_Mo_0.09_W_0.11_O_3−_ *_δ_* (BSM9W11), BaSc_0.8_Mo_0.11_W_0.09_O_3−_ *_δ_* (BSM11W9), and BaSc_0.8_Mo_0.15_W_0.05_O_3−_ *_δ_* (BSM15W5) (Table S1, Methods in the Supporting Information). Here, *δ* represents the amount of oxygen vacancies. X‐ray powder diffraction (XRD) measurements indicated that the as‐prepared BSM9W9 contained the main cubic perovskite phase with a small amount of Ba_3_Sc_4_O_9_ impurity (Figure 1e). In contrast, all reflections of other seven compositions were indexed by a primitive cubic cell, indicating the formation of single‐phase cubic perovskites (Figure 1f–l).

Impedance measurements were carried out in wet air to evaluate the bulk conductivity (*σ*_b_) of the eight compositions where the water vapor partial pressure *P*(H_2_O) was 0.021 atm. Representative impedance spectra are shown in Figures S1–S8. Spectra from all eight compositions revealed both bulk and grain‐boundary responses (see Figures S1a, S2a, S3a, S4a, S4b, S5a, S6a, S7a, and S8a). The *σ*_b_ and grain‐boundary conductivity (*σ*_gb_) were extracted by equivalent circuit analysis using the models shown in Figure S9. We obtained good fitting results (Figures S1–S8), reasonable capacitance values (Table S2), and small Kramers–Kronig residuals (Figure S10), validating the conductivity values. The obtained *σ*_b_ was higher than the *σ*_gb_ (Figure S11). Seven compositions of the Mo/W co‐doped BaScO_2.5_ materials, BaSc_1−_ *_x_*_−_ *_y_* Mo*_x_* W*_y_* O_3−_ *_δ_* exhibited higher bulk conductivity than BaZr_0.8_Y_0.2_O_2.9_ (BZY; Ref.[32]) and BaCe_0.9_Y_0.1_O_2.95_ (BCY; Ref.[25]). The higher bulk conductivity was attributed to the lower activation energy of BaSc_1−_ *_x_*_−_ *_y_* Mo*_x_* W*_y_* O_3−_ *_δ_* compared with BZY and BCY (e.g., at 100 °C, 0.38 eV for BSM10W10, 0.49 eV for BZY, 0.54 eV for BCY). Six of these compositions, BSM10W10, BSM5W15, BSM9W11, BSM11W11, BSM15W5, and BSM9W9 exhibited higher *σ*_b_ than the mono‐doped materials, BaSc_1−_ *_x_* Mo*_x_* O_3−_ *_δ_* (*x* = 0.2, 0.25 in Ref.[23] *x* = 0.225 in Ref.[43]) and BaSc_1−_ *_x_* W*_x_* O_3−_ *_δ_* (*x* = 0.2 [Ref.[40]], *x *= 0.25 [this work]) below 225 °C (Figure 1m,n). This demonstrates that the mixing of Mo and W atoms at the Sc *B*‐site increases the proton conductivity. Similar mixing effects have been observed in oxide‐ion conductors and Li‐ion conductors.^[^ ^44^, ^45^, ^46^ ^]^ Notably, the BSM10W10 exhibited the highest *σ*_b_ among all compositions including BSM20 and BSW20 (Figure 1m). The BSM10W10 has a higher conductivity than BSM20 and BSW20 due to the higher pre‐exponential factor *A* value 24 × 10^4^ S K cm^−1^ for BSM10W10, compared to 1.0 × 10^4^ S K cm^−1^ for BSM20 and 2.5 × 10^4^ S K cm^−1^ for BSW20. The equation *σ*_b_ *T* = *A* exp(−*E*_a_/*kT*) was used to estimate the *A* values, where the *E*_a_, *k*, and *T* are the activation energy for *σ*_b_, the Boltzmann constant, and the absolute temperature, respectively. Our further studies will focus on BSM10W10, because it exhibits the highest *σ*_b_ among the eight compositions.

**Figure 2:**
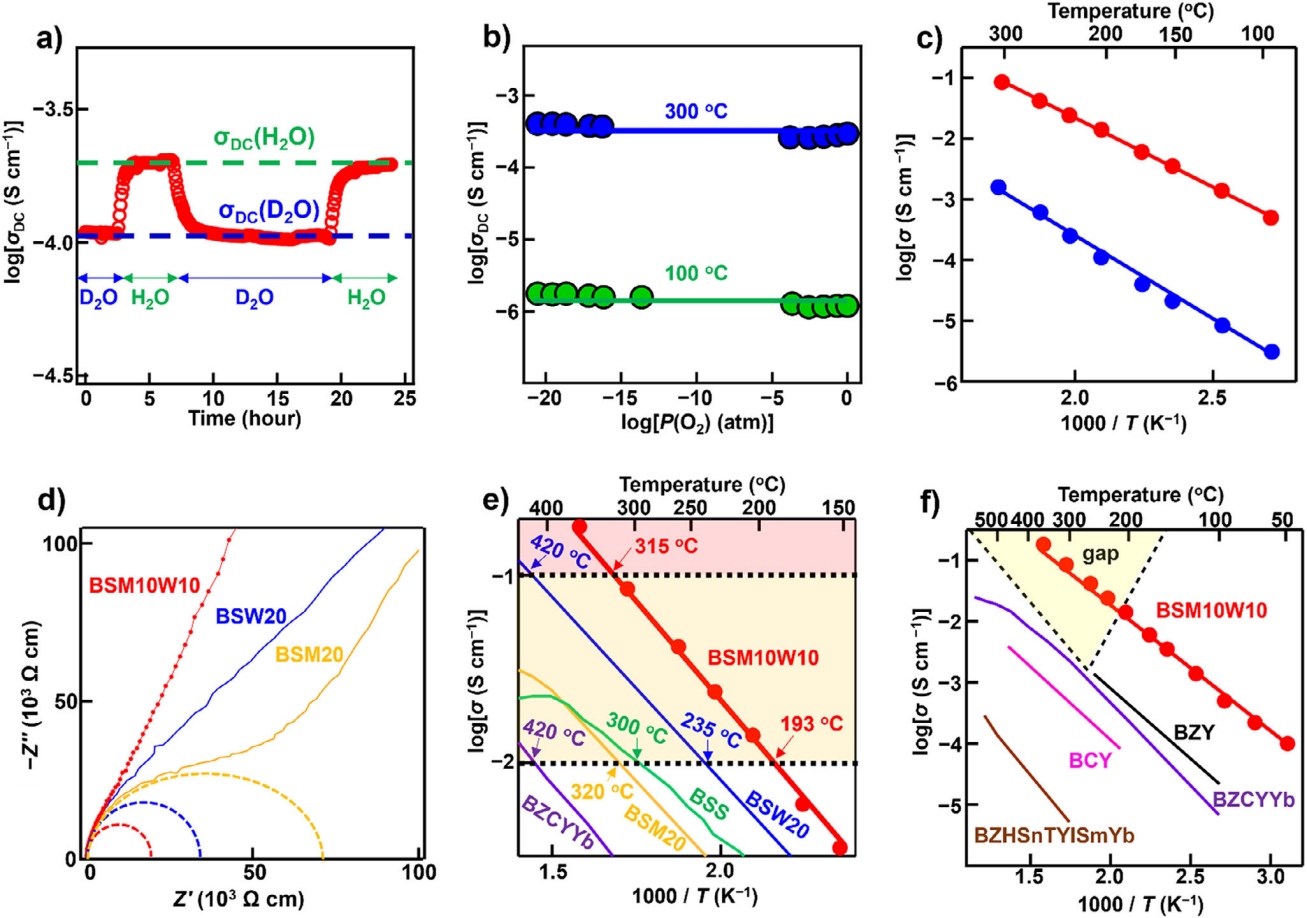
High proton conduction of BSM10W10. a) H/D isotope effect on the DC electrical conductivity *σ*_DC_ of BSM10W10 at 250 °C in wet air. b) Oxygen partial pressure dependencies of the *σ*_DC_ for BSM10W10 at 100 °C (green closed circles and solid line) and 300 °C (blue closed circles and solid line) under wet conditions. c) Arrhenius plots of bulk conductivity of BSM10W10 in wet air (*σ*_wet_, red closed circles and line) and dry air (*σ*_dry_, blue closed circles and line). d) Complex impedance plots (lines) and bulk component (dashed (depressed) semicircles) of BSM10W10 (red semicircle, closed circles, and line), BaSc_0.8_W_0.2_O_2.8_ (BSW20; blue semicircle and line),^[^ ^40^ ^]^ and BaSc_0.8_Mo_0.2_O_2.8_ (BSM20; yellow line and depressed semicircle)^[^ ^23^ ^]^ at 50 °C recorded in wet air. e) Arrhenius plots of bulk conductivity of BSM10W10 (this work), BSW20,^[^ ^40^ ^]^ BSM20,^[^ ^23^ ^]^ BaSc_0.75_Nb_0.25_O_2.75_ (BSN),^[^ ^41^ ^]^ BaSn_0.3_Sc_0.7_O_2.65_ (BSS),^[^ ^48^ ^]^ and BaZr_0.4_Ce_0.4_Y_0.1_Yb_0.1_O_2.9_ (BZCYYb).^[^ ^14^ ^]^ In the red and yellow regions, the conductivities are higher than 100 and 10 mS cm^−1^, respectively. f) Norby gap and Arrhenius plots of bulk conductivity of BSM10W10, acceptor mono‐doped proton conductors BaZr_0.8_Y_0.2_O_2.9_ (BZY)^[^ ^50^ ^]^ and BaCe_0.9_Y_0.1_O_2.95_ (BCY),^[^ ^25^ ^]^ and acceptor co‐doped proton conductors BaZr_1/8_Hf_1/8_Sn_1/8_Ti_1/8_Y_1/8_In_1/8_Sm_1/8_Yb_1/8_O_2.75_ (BZHSnTYISmYb)^[^ ^38^ ^]^ and BaZr_0.4_Ce_0.4_Y_0.1_Yb_0.1_O_2.9_ (BZCYYb).^[^ ^14^ ^]^

To demonstrate the proton conduction of BSM10W10, H/D isotope exchange experiments were performed at 250 °C in D_2_O‐ and H_2_O‐saturated air, where the water vapor partial pressure was 0.021 atm (Figure 2a). The direct current (DC) electrical conductivity (*σ*_DC_) of BSM10W10 was measured by the four‐probe method. First, *σ*_DC_ was measured under D_2_O‐saturated air. Then, during the *σ*_DC_ measurements, the atmosphere was switched from D_2_O‐saturated air to H_2_O‐saturated air. Next, the atmosphere was switched from H_2_O‐saturated air to D_2_O‐saturated air and then back again. The *σ*_DC_ in H_2_O‐saturated air, *σ*_DC_(H_2_O), was approximately 1.8 times higher than that in D_2_O‐saturated air, *σ*_DC_(D_2_O). The ratio of these two values, *σ*_DC_(H_2_O)/*σ*_DC_(D_2_O) = 1.8, is slightly higher than the expected value of 1.41 from the classical theory.^[^ ^47^ ^]^ This difference is likely due to the more significant impact of the zero‐point energy between a proton and a deuterium.^[^ ^47^, ^48^ ^]^ The *σ*_DC_ was almost independent of the oxygen partial pressure *P*(O_2_) in the wide *P*(O_2_) range between 10^−21^ and 1 atm at 300 and 100 °C under wet conditions (*P*(H_2_O) = 0.021 atm) (Figure 2b). This wide range indicates the wide proton conduction domain, as well as high chemical and electrical stability, suggesting ionic conduction. Warburg impedance was observed in the Nyquist plots of BSM10W10 at 204 °C and 303 °C (Figures S1c,d), which also suggests ion conduction. The bulk conductivity of BSM10W10 in wet air *σ*_wet_ was much higher than that in dry air *σ*_dry_ (e.g., *σ*_wet_ = 104 *σ*_dry_ at 200 °C; Figure 2c). To investigate the H/D isotope effect,^[^ ^47^ ^]^ the impedance measurements were performed on BSM10W10 in H_2_O/air and D_2_O/air. The difference in activation energy for bulk conductivity in H_2_O‐ and D_2_O‐saturated air *E*_D _− *E*_H_ was 0.04 eV (Table S3). Here, *E*_D_ and *E*_H_ are activation energies for bulk conductivity in D_2_O‐ and H_2_O‐saturated air, respectively. The activation energies *E*_a_ for the conductivities were estimated using the Arrhenius equation: *σ*_b_ *T* = *A* exp(−*E*_a_/*kT*). The value of *E*_D_−*E*_H_ 0.04 eV is close to 0.055 eV, which is predicted by the non‐classical theory.^[^ ^47^ ^]^ The ratio *A*_H_/*A*_D_ was 0.46, which is close to the ratios for other proton conductors.^[^ ^47^ ^]^ Here, *A*_D_ and *A*_H_ stand for the pre‐exponential factors in D_2_O‐ and H_2_O‐saturated air, respectively. These results indicate that protons are the dominant conducting species in BSM10W10.

Ceramic conductors exhibiting superprotonic conductivity higher than 0.1 S cm^−1^ at intermediate temperatures are extremely rare. In contrast, BSM10W10 showed the highest bulk proton conductivity *σ*_b_ among the ceramic proton conductors and superprotonic conduction within the “Norby gap” (e.g., 0.1 S cm^−1^ at 315 °C) (Figure 2d,e). It should be noted that the bulk conductivity *σ*_b_ of BSM10W10 in wet conditions reached 0.01 S cm^−1^ at 193 °C, which is much lower than 420 °C of BaZr_0.4_Ce_0.4_Y_0.1_Yb_0.1_O_2.9_ (BZCYYb) and 300 °C of BaSn_0.3_Sc_0.7_O_2.65_ (BSS; Ref.[48]). Furthermore, the *σ*_b_ of BSM10W10 reached 0.1 S cm^−1^ at 315 °C, which is 105 °C lower than 420 °C of BaSc_0.8_W_0.2_O_2.8_ (BSW20; Ref.[40]). BSM10W10 exhibited 190 times higher *σ*_b_ than BaCe_0.9_Y_0.1_O_2.95_ (BCY; Ref.[25]), 24 times higher *σ*_b_ than BaZr_0.8_Y_0.2_O_2.9_ (BZY; Ref.[32]), 7 times higher *σ*_b_ than BSS (Ref.[48]), and 3 times higher *σ*_b_ than BSW20 (Ref.[40]) at 220 °C. The high *σ*_b_ of BaSc_0.8_Mo_0.1_W_0.1_O_2.8−_ *_z_*_/2_(OH)*_z_* (BSM10W10) is attributable to the high proton concentration *z* and high diffusion coefficient, as discussed later. The proton conductivity of BSM10W10 created by donor co‐doping is much higher than those of acceptor co‐doped proton conductors such as BaZr_0.4_Ce_0.4_Y_0.1_Yb_0.1_O_2.9_ (BZCYYb) and BaZr_1/8_Hf_1/8_Sn_1/8_Ti_1/8_Y_1/8_In_1/8_Sm_1/8_Yb_1/8_O_2.75_ (BZHSTYISYb) (e.g., 73 times higher than BZCYYb at 100 °C, 16 000 times higher than BZHSTYISYb at 300 °C; Figure 2f).^[^ ^14^, ^35^, ^36^, ^38^ ^]^ This higher conductivity of BSM10W10 is attributed to the lower activation energy *E*_a_ for bulk conductivity of BSM10W10 (e.g., 0.38 eV at 100 °C) than that of an acceptor co‐doped oxides, BZCYYb (e.g., 0.58 eV at 100 °C). The *E*_a_ of the acceptor co‐doped oxide BZCYYb (e.g., 0.58 eV at 100 °C) was significantly higher than that of the acceptor mono‐doped oxide, BaZr_0.4_Ce_0.4_Y_0.2_O_2.9_ (BZCY) (e.g., 0.52 eV at 100 °C), which suggests the enhancement of proton trapping by acceptor co‐doping. The activation energy for proton diffusion coefficient of an isovalent Lu^3+^‐doped BaSc^3+^_0.8_Mo_0.2_O_2.8_ (BSLM: BaSc_0.6_Lu_0.2_Mo_0.2_O_2.8_) was higher than that of BaSc_0.8_Mo_0.2_O_2.8_, likely due to enhanced proton trapping (e.g., 0.48 eV for BSLM and 0.39 eV for BSM20 at 100 °C).^[^ ^23^, ^49^ ^]^ In sharp contrast, the *E*_a_ of BSM10W10 (e.g., 0.38 eV at 100 °C) created by donor co‐doping was comparable to those of donor mono‐doped proton conductors, BSM20 (e.g., 0.39 eV at 100 °C)^[^ ^23^ ^]^ and BSW20 (e.g., 0.39 eV at 100 °C),^[^ ^40^ ^]^ It is likely that the donor co‐doping does not increase the activation energy, resulting in higher proton conductivity compared with the acceptor co‐doping and isovalent doping (Figure 1a–d).

Electrochemical devices, such as PCFCs, require proton‐conducting electrolytes with high chemical stability for long‐term use. To investigate their chemical stability, the as‐prepared BSM10W10 powders were annealed under a CO_2_ flow at 250 °C for 24 h. No significant difference was observed between the XRD patterns before and after the annealing, demonstrating the material's exceptional stability against CO_2_ (Figure S12c,d). BSM10W10 powders were also stable during annealing in H_2_ and O_2_ at 250 °C for 24 h (Figure S12a,b). Taken together, the high proton conductivity, high chemical stability, and wide proton conduction domain indicate that BSM10W10 is an excellent proton conductor.

Next, we will explore the reasons for the high bulk proton conductivity (*σ*_b_) of BaSc_0.8_Mo_0.1_W_0.1_O_2.8−_ *_z_*_/2_(OH)*_z_* (BSM10W10). The *σ*_b_ is proportional to the proton concentration *z* in bulk BSM10W10: *σ*_b_ ∝ *z*, assuming the Nernst–Einstein equation:

(3) $$D=\sigma_{b}\mathrm{RTV}/F^{2}z$$

**Figure 3:**
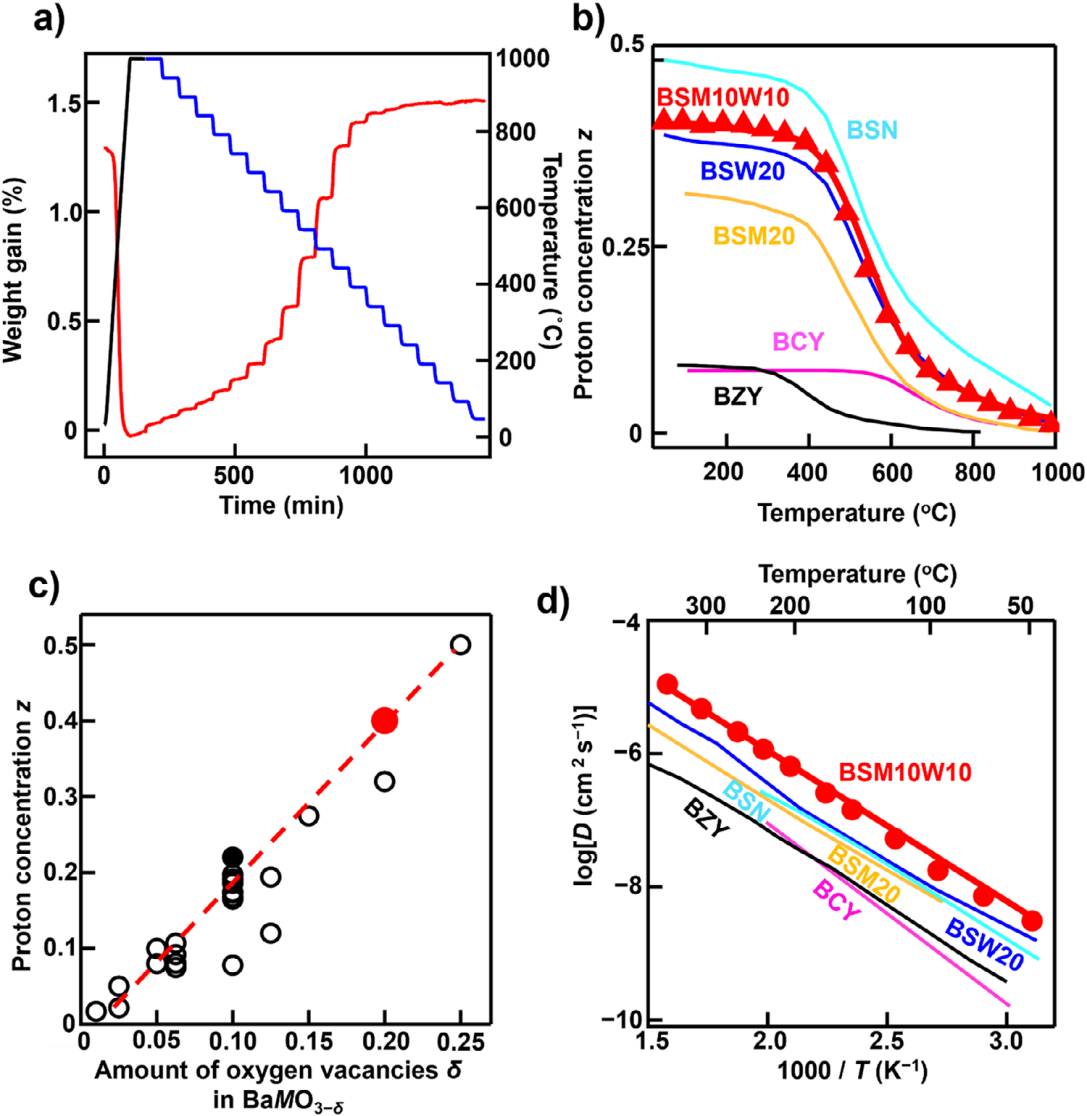
Origins of the high proton conductivity of bulk BSM10W10: the high proton concentration, a large amount of oxygen vacancies, and high diffusion coefficient. a) Water uptake of BSM10W10 in wet air. b) Temperature dependencies of proton concentration *z* in Ba*M*O_3−_ *_δ_*_−_ *_z_*_/2_(OH)*_z_* (red closed triangles and curve: BSM10W10 of this work, yellow curve: BSM20,^[^ ^23^ ^]^ blue curve: BSW20,^[^ ^40^ ^]^ light blue curve BSN,^[^ ^40^ ^]^ black curve: BZY,^[^ ^25^ ^]^ pink curve: BCY,^[^ ^25^ ^]^ which were obtained by TG measurements. Here, *M* represents the *B*‐site cations (e.g., *M* = Sc_0.8_Mo_0.1_W_0.1_ in BSM10W10). The hydration enthalpy and entropy of BSM10W10 are shown in Table S5. c) Correlation between the amount of oxygen vacancies *δ* in Ba*M*O_3−_ *_δ_* and *z* in Ba*M*O_3−_ *_δ_*_−_ *_z_*_/2_(OH)*_z_*. Red closed and black open circles stand for BSM10W10 (this work) and other Ba*M*O_3−_ *_δ_*_−_ *_z_*_/2_(OH)*_z_* from the literature.^[^ ^23^, ^25^, ^41^ ^]^ d) Arrhenius plots of experimental bulk proton diffusion coefficient *D* of Ba*M*O_3−_ *_δ_*_−_ *_z_*_/2_(OH)*_z_* (red circles and line: BSM10W10 (this work), blue line: BSW20,^[^ ^40^ ^]^ yellow line: BSM20,^[^ ^23^ ^]^ light blue line BSN,^[^ ^41^ ^]^ black line: BZY,^[^ ^25^ ^]^ and pink line: BCY^[^ ^25^ ^]^). The *D* values were estimated using Equation (3) and proton concentration *z* from TG measurements (Figure 3b).

Here, *σ*_b_ is the bulk conductivity, *R* is the gas constant, *V* is the lattice volume, *T* is the absolute temperature, *F* is the Faraday constant, and *z* is the proton concentration (number of protons in a unit cell). To examine the *z* and hydration of BSM10W10, we performed the thermogravimetric‐mass spectrometric (TG‐MS) and TG measurements (Figures S13, S14, and 3a). The TG‐MS results for the wet powders of BSM10W10 revealed that dehydration was the cause of weight loss during heating (Figure S13). Therefore, the *z* could be estimated from the weight change in the TG curve (Figure 3a). The TG data showed the typical hydration behavior and higher proton concentration *z* at lower temperatures (Figure 3b). At each temperature, the *z* value of BSM10W10 was higher than that of BSM20, BCY, and BZY (Figure 3b). For example, at 100 °C, *z* was 0.4 for BSM10W10, which was higher than 0.32 for BSM20, 0.1 for BCY, and 0.1 for BZY. Therefore, the higher proton concentration of BSM10W10 is a reason for its higher proton conductivity of BSM10W10 compared with BSM20, BCY, and BZY.

**Figure 4:**
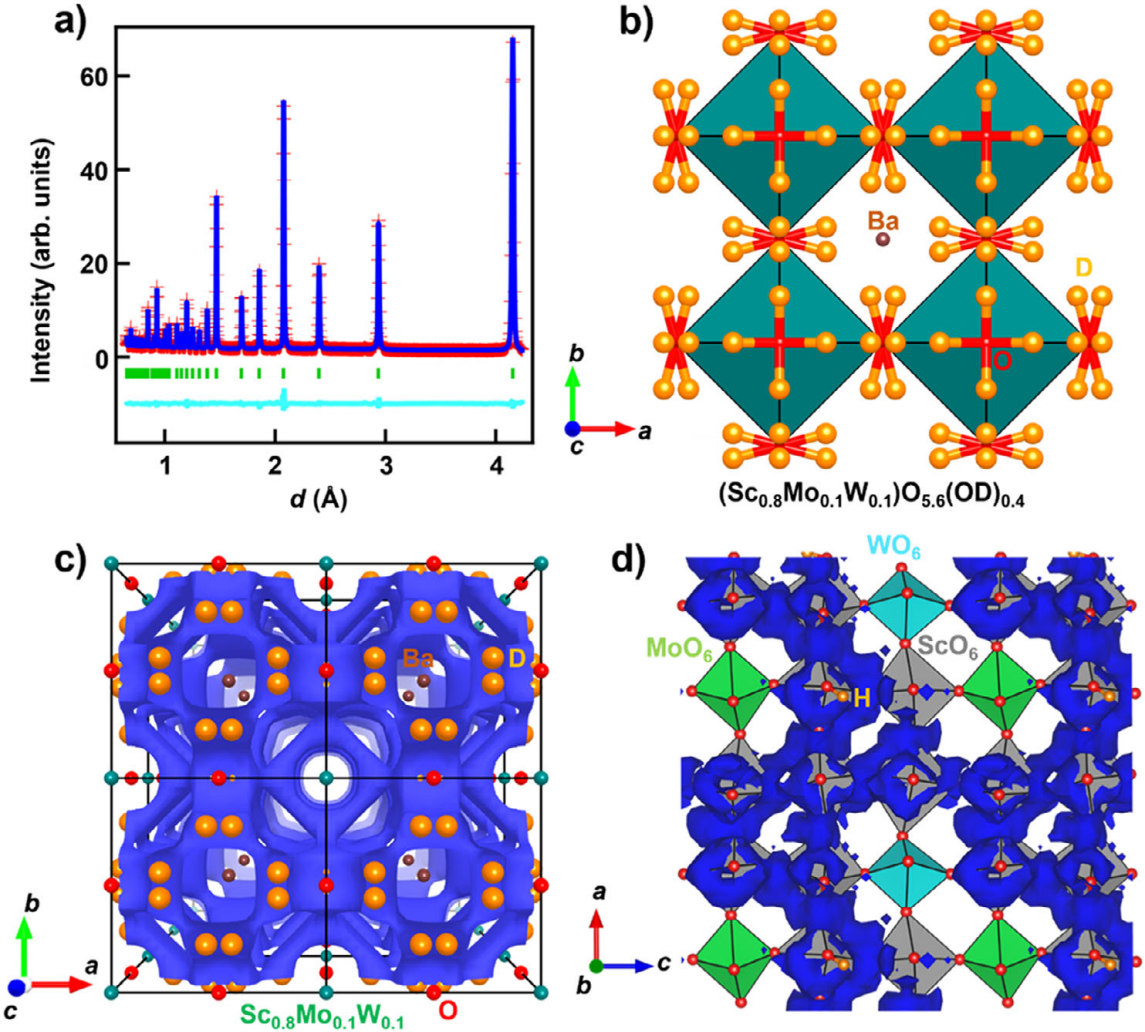
Structural origins of the high bulk proton conductivity of BSM10W10. a) Rietveld pattern of the neutron diffraction data of hydrated (deuterated) BSM10W10 pellets taken at −243 °C. Green tick marks denote the calculated Bragg peak positions of cubic $Pm\bar{3}m$ BSM10W10. Blue lines and red crosses are the calculated and observed intensities, respectively. The light blue line is the difference pattern. b) Refined crystal structure of BSM10W10 drawn with green (Sc_0.8_Mo_0.1_W_0.1_)O_5.6_(OD)_0.4_ octahedra, violet Ba, red O, and orange D atoms. The red/orange lines denote the OD bonds. The isotropic displacement spheres of Ba, O, and D atoms are plotted at the 30% probability level. The OD bond length calculated for the refined structure of BSM10W10 (0.966(3) Å) agreed with the OH bond length values estimated from the Raman scattering data (0.99(6) Å; Figure S17) and from the optimized structure by DFT calculations (1.01(9) Å; Figure S16) within two estimated standard deviations. These results indicate the formation of hydroxide ions OH in BaSc_0.8_Mo_0.1_W_0.1_O_2.8−_ *_z_*_/2_(OH)*_z_* and OD in BaSc_0.8_Mo_0.1_W_0.1_O_2.8−_ *_z_*_/2_(OD)*_z_*. c) Bond‐valence based energy landscape (BVEL) for a test proton with the blue isosurface at 0.39 eV, which was calculated for the crystal parameters refined using the neutron diffraction data of BSM10W10. Energy barrier for proton migration was estimated to be 0.35 eV from the BVEL, which was consistent with the experimental activation energy for bulk conductivity 0.38 eV. Black lines denote the unit cells. Ba, O, D, and Sc_0.8_Mo_0.1_W_0.1_ atoms are denoted by the brown, red, orange, and green spheres, respectively. d) Blue isosurface of the proton probability density at 0.0005 Å^−3^ in Ba_27_Sc_23_Mo_2_W_2_O_81_H_15_ viewed along the *b* axis from the AIMD simulations at 1500 °C (−0.2 ≤ *x* ≤ 1.5; 0.93 ≤ *y* ≤ 1.21; −0.2 ≤ *z* ≤ 1.5). The red and orange spheres represent O and H atoms, respectively. The green, light blue, and gray squares denote MoO_6_, WO_6_ and ScO_6_ octahedra, respectively.

In general, the proton concentration (*z*) determined from the TG measurements contains contributions not only from the bulk but also from the grain boundaries and surfaces. To clarify the contributions, the *z*, and hydration of bulk BSM10W10, we performed Rietveld refinements of neutron diffraction data of a hydrated (deuterated) BaSc_0.8_Mo_0.1_W_0.1_O_2.8−_ *_z_*_/2_(OD)*_z_* pellet (BSM10W10) at −243 °C (Figure 4, Table S4; See the details in the Supplementary Note of Supporting Information). In a preliminary analysis, the occupancy factor of the oxygen atom was refined to be 1.0000(3), indicating the full hydration, in which the oxygen site was fully occupied by oxygen atoms. In a separate analysis, the refined proton (deuteron) occupancy factor yielded the proton concentration value *z* = 0.4001(17), in excellent agreement with the value *z* = 0.4 from the TG results. Thus, both structural and TG analyses consistently demonstrate the full hydration in bulk BSM10W10. These results indicate that the hydrated (deuterated) composition of BSM10W10 is BaSc_0.8_Mo_0.1_W_0.1_O_3.0_D_0.4_, showing the full hydration. The full hydration is one of the reasons for high proton concentration *z*. The high bulk proton concentration 0.4 of BaSc_0.8_Mo_0.1_W_0.1_O_3.0_D_0.4_ is one of the reasons for the high bulk conductivity. Another reason for the high proton concentration *z* in BSM10W10 is the large amount of oxygen vacancies (*δ* = 0.2) in dry BaSc_0.8_Mo_0.1_W_0.1_O_3−_ *_δ_* without water. Indeed, the *z* of numerous hydrated perovskites, Ba*M*O_3−_ *_δ_*_−_ *_z_*_/2_(OH)*_z_*, increases with increasing *δ* in dry Ba*M*O_3−_ *_δ_* materials without water (Figure 3c). Here, *M* represents the *B*‐site cations (e.g., *M* = Sc_0.8_Mo_0.1_W_0.1_ in BSM10W10).

The proton concentration *z* of BSM10W10 is nearly equal to that of BSW20 and lower than that of BSN. However, BSM10W10 had a higher bulk proton conductivity than BSW20 and BSN. These results indicate that the proton diffusion coefficient of BSM10W10 is higher than those of BSW20 and BSN. We estimated the bulk proton diffusion coefficient *D* of BSM10W10 using the Nernst–Einstein equation (Equation (3) and the proton concentration *z* obtained from the TG measurements. The estimated *D* value of BSM10W10 was higher than those of BSW20, BSM20, BSN, BZY, and BCY (Figure 3d). From Equation (3), the bulk conductivity *σ*_b_ can be expressed as *σ*_b _= *DF* ^2^ *z*/*RTV*. Since both *D* and *z* of BSM10W10 are high (Figure 3b,d), the *σ*_b_ of BSM10W10 is higher than BSM20, BZY, and BCY. The reason for higher proton conductivity of BSM10W10 than BSW20 and BSN is high bulk proton diffusion coefficient *D* of BSM10W10.

Figure 4c shows the isosurface of bond‐valence‐based energy for a test proton of BSM10W10, indicating three‐dimensional network of proton diffusion pathway. The three‐dimensional proton diffusion might be beneficial for the polycrystalline electrolytes in electrochemical devices.^[^ ^40^ ^]^ The snapshots from ab initio molecular dynamics (AIMD) simulations showed Grotthuss‐type proton conduction mechanism in BSM10W10 where the protons migrate via the rotation around an oxide ion and hopping between adjacent oxide ions (Figure S15, movie). The AIMD simulations also demonstrated that most of protons are not near the oxide ions coordinated to a W or Mo donor dopant but to two Sc cations (Figure 4d). This result indicates that the protons migrate around ScO_6_ octahedra while avoiding the MoO_6_ and WO_6_ octahedra, supporting the schematic energy diagram in Figure 1d. Similar proton migrations have been observed in Mo‐, W‐, and Nb‐doped BaScO_2.5_, as reported by Saito et al.^[^ ^23^, ^40^, ^41^ ^]^ Recently, Tsujikawa et al. demonstrated the similar proton migration around ScO_6_ octahedra.^[^ ^48^ ^]^ The Sc occupancy at the *B* site of BSM10W10 is as high as 0.8. This high Sc occupancy forms a connected network of ScO_6_ octahedra, which leads to the high proton mobility.

**Figure 5:**
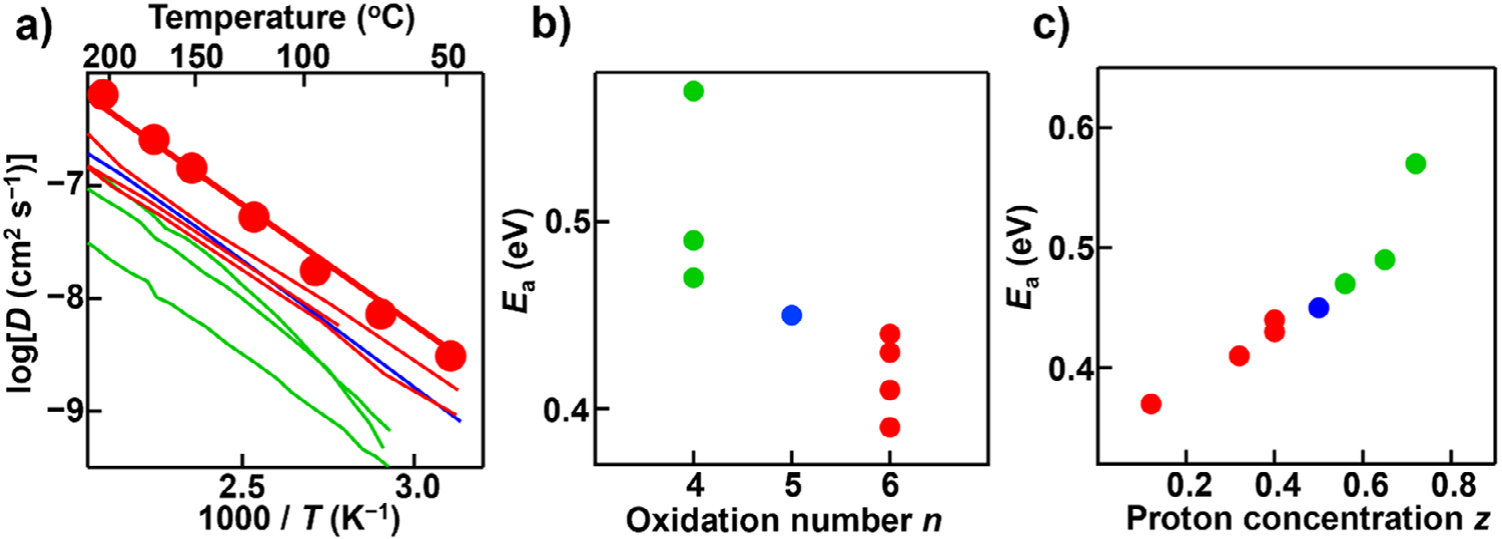
a) Arrhenius plots of the diffusion coefficient *D* of BaSc_1−_ *_x_M^n^* ^+^ *_x_* O_3−_ *_δ_*_−_ *_z_*_/2_(OH)*_z_* (*n* = 6 for *M* = Mo/W, Mo, and W; *n* = 5 for Nb; *n* = 4 for Ti, Zr, and Sn).^[^ ^23^, ^40^, ^41^, ^48^, ^51^ ^]^ Here, *M^n^* ^+^ is the donor dopant with a higher oxidation number than the host cation, *n* is the oxidation number of the *M^n^* ^+^ cation (*n* > 3), *x* is the dopant concentration, *δ* is the amount of oxygen vacancies, *z* is the proton concentration. Red circles denote the data of BaSc_0.8_Mo_0.1_W_0.1_O_2.8−_ *_z_*_/2_(OH)*_z_* (BSM10W10). b) Plots of *n* versus activation energy *E*_a_ for *D* at 70−170 °C. c) Plots of *z* versus activation energy *E*_a_ for *D* at 70−170 °C. Red, blue, and green circles and lines denote the data of BaSc_1−_ *_x_M* ^6+^ *_x_* O_3−_ *_δ_*_−_ *_y_*_/2_(OH)*_z_* (*n* = 6), BaSc_1−_ *_x_M* ^5+^ *_x_* O_3−_ *_δ_*_−_ *_y_*_/2_(OH)*_z_* (*n* = 5), and BaSc_1−_ *_x_M* ^4+^ *_x_* O_3−_ *_δ_*_−_ *_y_*_/2_(OH)*_z_* (*n* = 4), respectively.

As described above, BSM10W10 (*n* = 6) exhibited higher bulk proton conductivity compared with BaSc_1−_ *_x_M* ^4+^ *_x_* O_3−_ *_δ_*_−_ *_z_*_/2_(OH)*_z_* (*n* = 4) such as BSS. Here, *M^n^* ^+^ is the donor dopant with a higher oxidation number than the host cation, *n* is the oxidation number of the *M^n^* ^+^ cation, *x* is the dopant concentration, and *δ* is the amount of oxygen vacancies. To investigate its reasons, we compared the conductivity diffusion coefficient *D* of BaSc_1−_ *_x_M^n^* ^+^ *_x_* O_3−_ *_δ_*_−_ *_z_*_/2_(OH)*_z_* (*M* = Mo/W, Mo, W, Nb, Ti, Zr, Sn). The *D* of BaSc_1−_ *_x_M* ^6+^ *_x_* O_3−_ *_δ_*_−_ *_z_*_/2_(OH)*_z_* (*n* = 6) was higher than those of BaSc_1−_ *_x_M* ^4+^ *_x_* O_3−_ *_δ_*_−_ *_z_*_/2_(OH)*_z_* (*n* = 4) below 170 °C (Figure 5a). Therefore, the higher proton conductivity of *n* = 6 was attributable to this higher *D* of *n* = 6 compared to *n* = 4. This higher *D* of *n* = 6 can be attributed to lower activation energy *E*_a_ for *D* of *n* = 6 compared with *n* = 4 (Figure 5b). The *E*_a_ for *D* increased with an increase of proton concentration *z* in BaSc_1−_ *_x_M^n^* ^+^ *_x_* O_3−_ *_δ_*_−_ *_z_*_/2_(OH)*_z_* (Figure 5c). Thus, the higher *E*_a_ of *n* = 4 can be ascribed to higher proton concentration, indicating the overdoping. Similar overdoping was observed in Y‐doped BaZrO_3_.^[^ ^32^ ^]^ The moderate proton concentration of *n* = 6 can lead to low *E*_a_ for *D* and high *D*, resulting in high bulk proton conductivity.

## Conclusion

In conclusion, we have discovered a new series of Mo/W donor co‐doped BaScO_2.5_ materials, BaSc_1−_ *_x_*_−_ *_y_* Mo*_x_* W*_y_* O_3−_ *_δ_* ( = BaSc_1−_ *_x_*_−_ *_y_* Mo*_x_* W*_y_* O_2.5+3_ *_x_*_/2+3_ *_y_*_/2−_ *_z_*_/2_(OH)*_z_*). Seven compositions of BaSc_1−_ *_x_*_−_ *_y_* Mo*_x_* W*_y_* O_3−_ *_δ_*, exhibit higher bulk proton conductivity than the acceptor‐doped BaZrO_3_‐based materials such as BaZr_0.8_Y_0.2_O_2.9_ (BZY) and acceptor co‐doped oxides as BaZr_0.4_Ce_0.4_Y_0.1_Yb_0.1_O_2.9_ (BZCYYb). The higher conductivity is attributable to the lower activation energy for bulk proton conductivity in BaSc_1−_ *_x_*_−_ *_y_* Mo*_x_* W*_y_* O_3−_ *_δ_* compared with acceptor‐doped proton conductors. Five compositions of BaSc_1−_ *_x_*_−_ *_y_* Mo*_x_* W*_y_* O_3−_ *_δ_* exhibit higher proton conductivity than the mono‐doped compositions BaSc_1−_ *_x_* Mo*_x_* O_3−_ *_δ_* and BaSc_1−_ *_y_* W*_y_* O_3−_ *_δ_*, indicating the enhancement of the proton conductivity by the mixing of Mo and W at the Sc *B*‐site. In particular, we have demonstrated that BSM10W10 (BaSc_0.8_Mo_0.1_W_0.1_O_2.8_) exhibits the highest proton conductivity among BaSc_1−_ *_x_*_−_ *_y_* Mo*_x_* W*_y_* O_3−_ *_δ_* and known ceramic proton conductors (e.g., 0.1 S cm^−1^ at 315 °C and 0.01 S cm^−1^ at 193 °C). The BSM10W10 shows high chemical stability under CO_2_, O_2_, and H_2_ atmospheres. The high proton conductivity of BSM10W10 is due to the following reasons: 1) The high proton concentration resulting from the large amount of oxygen vacancies in dry material and full hydration. 2) The high proton diffusion coefficient *D* due to the low activation energy for *D* and/or the high pre‐exponential factor for *D* and the high Sc content (80 mol% at the *B*‐site) forming the network of ScO_6_ octahedra for proton diffusion. We have also demonstrated that the proton conductivity of Mo/W donor co‐doped materials surpasses that of the acceptor co‐doped oxides and the isovalent cation doped material. This higher conductivity is likely due to the reduced proton trapping in donor co‐doped materials, resulting in a lower activation energy compared to acceptor co‐doped and isovalent cation doped materials. The present work has also demonstrated that the activation energy for *D* of BaSc_1−_ *_x_M^n^* ^+^ *_x_* O_3−_ *_δ_*_−_ *_z_*_/2_(OH)*_z_* (*M* = Mo/W, Mo, W, Nb, Ti, Zr, Sn) increases with an increase of the proton concentration and shows a decrease trend with an increase of the oxidation number of donor dopant *M* (*n*), leading to higher proton conductivity of materials for *n* = 6 compared with for *n* = 4. These results highlight the potential of a novel strategy for developing superprotonic conductors at intermediate temperatures: “donor co‐doping into oxides with intrinsic oxygen vacancies” and “doping of donor with high oxidation number (e.g., *n* = 6) into oxides with intrinsic oxygen vacancies”. The BSM10W10 exhibits high bulk conductivity and lower total conductivity than bulk conductivity due to the large grain boundary resistance. Grain growth is known to increase the total conductivity of ceramic proton conductors.^[^ ^52^ ^]^ Thus, the grain growth of BSM10W10 would be effective to improve its total conductivity, leading to high performance of the electrochemical devices using the BSM10W10 materials. These findings of high proton conductors and this strategy open new avenues for innovative proton conductors created by donor co‐doping at intermediate temperatures.

## Supporting Information

We have cited additional references within the Supporting Information.^[^ ^53^, ^54^, ^55^, ^56^, ^57^, ^58^, ^59^, ^60^, ^61^, ^62^, ^63^, ^64^, ^65^ ^]^

## Conflict of Interests

The authors declare no conflict of interest.

## Supporting information



Supporting information

Supporting information

## Data Availability

The data that support the findings of this study are available in the Supporting Information of this article.
