# Supplementary material for: Superprotonic Conduction in Donor Co‐Doped Perovskites
Source: Angew Chem Int Ed Engl. 2026 Jan 19;65(9):e21773. doi: 10.1002/anie.202521773 (PMC12930018; doi:10.1002/anie.202521773)
Supplement: Supplementary file 1 — Supporting information [file ANIE-65-e21773-s002.pdf]

Supporting Information

Superprotonic Conduction in Donor Co-Doped Perovskites

Kensei Umeda[a],+, Kei Saito[a],+, Takashi Honda[b,c], Masatomo Yashima[a,d],*

[a] Mr. K. Umeda, Dr. K. Saito, Prof. M. Yashima

Department of Chemistry, School of Science

Institute of Science Tokyo

2-12-1-W4-17, O-okayama, Meguro-ku, Tokyo 152-8551, Japan

[b] Dr. T. Honda

Institute of Materials Structure Science

High Energy Accelerator Research Organization (KEK)

Tsukuba, Ibaraki, 305-0801, Japan

[c] Dr. T. Honda

J-PARC Center

High Energy Accelerator Research Organization (KEK)

Tokai, Ibaraki, 319-1106, Japan

[d] Prof. M. Yashima

Center for Energy Systems Design (CESD)

International Institute for Carbon Neutral Energy Research (WPI-I^{2}CNER), Kyushu University

744 Motooka, Nishi-ku, Fukuoka 819-0395, Japan.

* Corresponding author E-mail: yashima@chem.sci.isct.ac.jp

+ Both authors contributed equally to this work.

Methods

$BaSc_{1-x-y}Mo_xW_yO_{3-\delta}$ (= $BaSc_{1-x-y}Mo_xW_yO_{2.5+3x/2+3y/2-z/2}(OH)_z$) samples were synthesized by solid-state reactions where the *x* and *y* are the concentration of Mo and W, respectively, and *z* is the proton concentration. We refer $BaSc_{1-x-y}Mo_xW_yO_{3-\delta}$ to the abbreviations as shown in Table S1. The raw materials $BaCO_3$ (99.95%, Kojundo Chemical Laboratory Co., Ltd., Japan), Sc_2O_3 (Shin-Etsu Chemical Co., 99.99%), MoO_3 (Kojundo Chemical Laboratory Co., 99.99%), and WO_3 (Kojundo Chemical Laboratory Co., 99.99%) were mixed and ground as ethanol slurries and dry powders in an agate mortar for about 1 h. The mixed powders were calcined in air at 900 °C for 12 h to remove the carbonates. The calcined powders were ground into fine powders in the agate mortar for about 1h as ethanol slurries and dry powders. The fine powders were uniaxially pressed into pellets at about 150 MPa and then sintered in air at 1600 °C for 10 h. The sintered pellets were crushed in a tungsten carbide crusher and ground in the agate mortar for about 1 h as ethanol slurries and dry powders. The obtained samples were uniaxially pressed into pellets at about 150 MPa, isostatically pressed into pellets at approximately 200 MPa, and sintered in air at 1600 °C for 10–20 h. We refer the sintered products to "as-sintered pellets". The relative densities of the as-sintered pellets were 65–75%. The as-sintered pellets of BSM9W9, BSM10W10, BSM11W11, BSM5W15, BSM9W11, BSM11W9, BSM15W5, and BSW25 were used for the alternative current (AC) impedance and direct current (DC) electrical conductivity measurements. Parts of the sintered products were crushed and ground into fine powders to carry out X-ray powder diffraction (XRD), X-ray fluorescence (XRF), inductively coupled plasma optical emission spectroscopy (ICP-OES) and thermogravimetric (TG) measurements. We refer the powders thus obtained to "as-prepared powders". The chemical composition of BSM10W10 was determined to be Ba: Sc: Mo: W = 1.0: 0.8: 0.1: 0.1 by XRF analysis (NEX DE, Rigaku Co.), which was in good agreement with that of nominal composition. The atomic ratios of the as-prepared BSM10W10 and BSM9W9 powders were determined to be Ba: Sc: Mo: W = 1.0: 0.8: 0.1: 0.1 and 1.0: 0.82: 0.09: 0.09, respectively, by ICP-OES analyses (SPS3500DD, Hitachi High-Tech Co.). Cu Kα XRD data of as-prepared powders of BSM9W9, BSM10W10, BSM11W11, BSM5W15, BSM9W11, BSM11W9, BSM15W5, and BSW25 samples were measured at 24 °C with a laboratory-based X-ray diffractometer (MiniFlex, Rigaku Co.). Scanning electron microscope (SEM) observation of an as-sintered pellet of BSM10W10 was performed using a VE-8800 SEM microscope (Keyence Co.) (Fig. S18). The proton concentration of BSM10W10 was investigated from 1000 to 100 °C by TG analysis (STA449 Jupiter, Netzsch Co.) In the TG measurements, the as-prepared powders of BSM10W10 were heated to 1000 °C in dry air (water vapor pressure $P(H_2O) < 10^{-4}$ atm) at the heating rate of 10 °C min^{-1} and kept at 1000 °C for 1 h in dry air to remove the water and carbonates. The gas was subsequently switched to wet air ($P(H_2O)$ = 0.021 atm). The water vapor pressure $P(H_2O)$

was controlled by a water bath. In cooling process, the samples were kept for 1 h at each temperature of 1000, 950, 900, 850, 800, 750, 700, 650, 600, 550, 500, 450, 400, 350, 300, 250, 200, 150, 100, and 50 °C. The proton concentration z in $BaSc_{0.8}Mo_{0.1}W_{0.1}O_{2.8-z/2}(OH)_z$ was calculated from the weight increase assuming that the weight gain is due to the water incorporation only and that the sample contained no protons (z = 0) at 1000 °C in dry air. The hydration enthalpy and entropy were estimated using the TG data (Table S5).

To measure the thermogravimetric TG-MS and Raman scattering data, “wet powders” of BSM10W10 were prepared as follows. The as-prepared powders of BSM10W10 were heated up to 1000 °C and kept at 1000 °C for 2 h in wet air ($P(H_2O)$ = 0.021 atm). In cooling process, the sample was kept for 1 h at 900, 800, 700, 600, 500, 400, 300, 200, 100, and 50 °C to reach equilibrium. The powders thus obtained are called as “wet powders”. TG-MS analyses of wet powders of BSM10W10 were performed under dry He ($P(H_2O) < 1.5\times10^{-4}$ atm) flow at a heating rate of 20 °C min^{-1} up to 1000 °C using a Thermo Mass Photo system (Rigaku Co., Ltd.). Raman spectrum of the wet powders of BSM10W10 was collected with NRS-4100 (JASCO Co.) with excitation wavelength of 532 nm.

Impedance spectra of the as-sintered pellets of BSM9W9, BSM10W10, BSM11W11, BSM5W15, BSM9W11, BSM11W9, BSM15W5, and BSW25 (4.5–4.8 mm in diameter, 8–10 mm in thickness, Fig. S19) with Pt electrodes were recorded on cooling in wet air ($P(H_2O)$ = 0.021 atm) and dry air ($P(H_2O) < 1.5\times10^{-4}$ atm) with a Solartron 1260 impedance analyzer in the frequency range from 0.1 Hz to 10 MHz with an applied alternating voltage of 100 mV. Equivalent-circuit analyses were performed to extract the bulk conductivity and grain-boundary conductivity using *ZView* software (Scribner Associates, Inc.). The *Lin–KK* software was employed to perform the Kramers–Kronig transformation on the collected impedance data.[53-55] The direct current (DC) electrical conductivity of BSM10W10 was measured with Pt electrodes by the DC four-probe method. The isotope effect of as-sintered pellet of BSM10W10 was evaluated by the DC electrical conductivity measurements at 250 °C in D_2O- and H_2O-saturated air (water vapor pressure $P(D_2O) = P(H_2O)$ = 0.021 atm). Oxygen partial pressure $P(O_2)$ dependence of the DC electrical conductivity of as-sintered pellet of BSM10W10 (4.5 mm in diameter and 14 mm in length) was investigated at 300 °C and 100 °C using a mixture of O_2, air, N_2 and 5% H_2 in N_2 under wet conditions ($P(H_2O)$ = 0.021 atm), where the $P(O_2)$ was monitored with an oxygen sensor placed at the outlet of the apparatus.

Neutron diffraction data of the hydrated (deuterated) $BaSc_{0.8}Mo_{0.1}W_{0.1}O_{2.8-z/2}(OD)_z$ (BSM10W10) pellets were measured at –243 °C with time-of-flight neutron diffractometer NOVA at the MLF of the J-PARC.[56] The hydrated pellets of BSM10W10 for the neutron diffraction measurements were prepared as follows. The as-sintered pellets of

BSM10W10 were heated to 1000 °C in dry air flow ($P(H_2O) < 1.5\times10^{-4}$ atm) and kept for 1h to dehydrate, and then the dry air flow was switched to D_2O-saturated air flow ($P(D_2O)$ = 0.021 atm) at the same temperature 1000 °C. In cooling process, the sample was kept for 2h at 1000, 900, 800, 700, 600, 500, 400, 300, 200, 100, and 50 °C to reach equilibrium. Rietveld analyses of the hydrated BSM10W10 pellets were performed with *Z-Rietveld*[57,58] using the neutron diffraction data taken with the backscattering bank of the NOVA. The bond-valence-based-energy landscape (BVEL) for a test proton in BSM10W10 was calculated using its refined crystal parameters at −243 °C with the *SoftBV* program.[59,60] The refined structures and BVEL were depicted using the *VESTA 3*.[61]

The static DFT calculations were performed for the 3×3×3 supercells $Ba_{27}Sc_{23}Mo_2W_2O_{81}H_{15}$ (~$[BaSc_{0.8}Mo_{0.1}W_{0.1}O_3H_{0.4}]_{27}$ with the Vienna *ab initio* Simulation Package (VASP) code with the projector augmented wave (PAW) method and the Perdew-Burke-Ernzerhof (PBE) functional in the generalized gradient approximation (GGA). The cut-off energy was set to 400 eV for all the calculations. A 3×3×3 set of *k*-point meshes was used in Monkhorst-Pack scheme. First, we optimized 30 structural models of $Ba_{27}Sc_{23}Mo_2W_2O_{81}$ using the background charge. Next, the structures of $Ba_{27}Sc_{23}Mo_2W_2O_{81}H_{15}$ were optimized using the models with lowest energies of $Ba_{27}Sc_{23}Mo_2W_2O_{81}$. Here, in the initial models, each proton was put near the oxygen atom where the O-H distance was approximately 1 Å. The AIMD simulations were performed at a constant temperature 1500 °C with a time step of 0.5 fs within the canonical ensemble (NVT) using a Nosé thermostat, after the heating process (1 °C fs^{-1}) within the micro-canonical (NVE) ensemble. The probability density distribution of H atoms from the AIMD simulations were drawn with *VESTA 3*.[61] To visualize the AIMD snapshots and trajectories, we used the OVITO program.[62]

Table S1. Abbreviations of the chemical compositions. Same abbreviation is used for both dry samples without water $BaSc_{1-x-y}Mo_xW_yO_{2.5+3x/2+3y/2}$ and hydrated samples $BaSc_{1-x-y}Mo_xW_yO_{2.5+3x/2+3y/2-z/2}(OH)_z$.

Abbreviation	Composition of dry sample without water	Composition of hydrated samples $BaSc_{1-x-y}Mo_xW_yO_{2.5+3x/2+3y/2-z/2}(OH)_z$	x	y	References
BSM9W9	$BaSc_{0.82}Mo_{0.09}W_{0.09}O_{2.77}$	$BaSc_{0.82}Mo_{0.09}W_{0.09}O_{2.77-z/2}(OH)_z$	0.09	0.09	This work
BSM10W10	$BaSc_{0.8}Mo_{0.1}W_{0.1}O_{2.8}$	$BaSc_{0.8}Mo_{0.1}W_{0.1}O_{2.8-z/2}(OH)_z$	0.1	0.1	This work
BSM11W11	$BaSc_{0.78}Mo_{0.11}W_{0.11}O_{2.83}$	$BaSc_{0.78}Mo_{0.11}W_{0.11}O_{2.83-z/2}(OH)_z$	0.11	0.11	This work
BSW25	$BaSc_{0.75}W_{0.25}O_{2.875}$	$BaSc_{0.75}W_{0.25}O_{2.875-z/2}(OH)_z$	0	0.25	This work
BSW20	$BaSc_{0.8}W_{0.2}O_{2.8}$	$BaSc_{0.8}W_{0.2}O_{2.8-z/2}(OH)_z$	0	0.20	[40]
BSM5W15	$BaSc_{0.8}Mo_{0.05}W_{0.15}O_{2.8}$	$BaSc_{0.8}Mo_{0.05}W_{0.15}O_{2.8-z/2}(OH)_z$	0.05	0.15	This work
BSM9W11	$BaSc_{0.8}Mo_{0.09}W_{0.11}O_{2.8}$	$BaSc_{0.8}Mo_{0.09}W_{0.11}O_{2.8-z/2}(OH)_z$	0.09	0.11	This work
BSM11W9	$BaSc_{0.8}Mo_{0.11}W_{0.09}O_{2.8}$	$BaSc_{0.8}Mo_{0.11}W_{0.09}O_{2.8-z/2}(OH)_z$	0.11	0.09	This work
BSM15W5	$BaSc_{0.8}Mo_{0.15}W_{0.05}O_{2.8}$	$BaSc_{0.8}Mo_{0.15}W_{0.05}O_{2.8-z/2}(OH)_z$	0.15	0.05	This work
BSM20	$BaSc_{0.8}Mo_{0.2}O_{2.8}$	$BaSc_{0.8}Mo_{0.2}O_{2.8-z/2}(OH)_z$	0.20	0	[23]
BSM22.5	$BaSc_{0.775}Mo_{0.225}O_{2.8375}$	$BaSc_{0.775}Mo_{0.225}O_{2.8375-z/2}(OH)_z$	0.225	0	[43]
BSM25	$BaSc_{0.75}Mo_{0.25}O_{2.875}$	$BaSc_{0.75}Mo_{0.25}O_{2.875-z/2}(OH)_z$	0.25	0	[23]

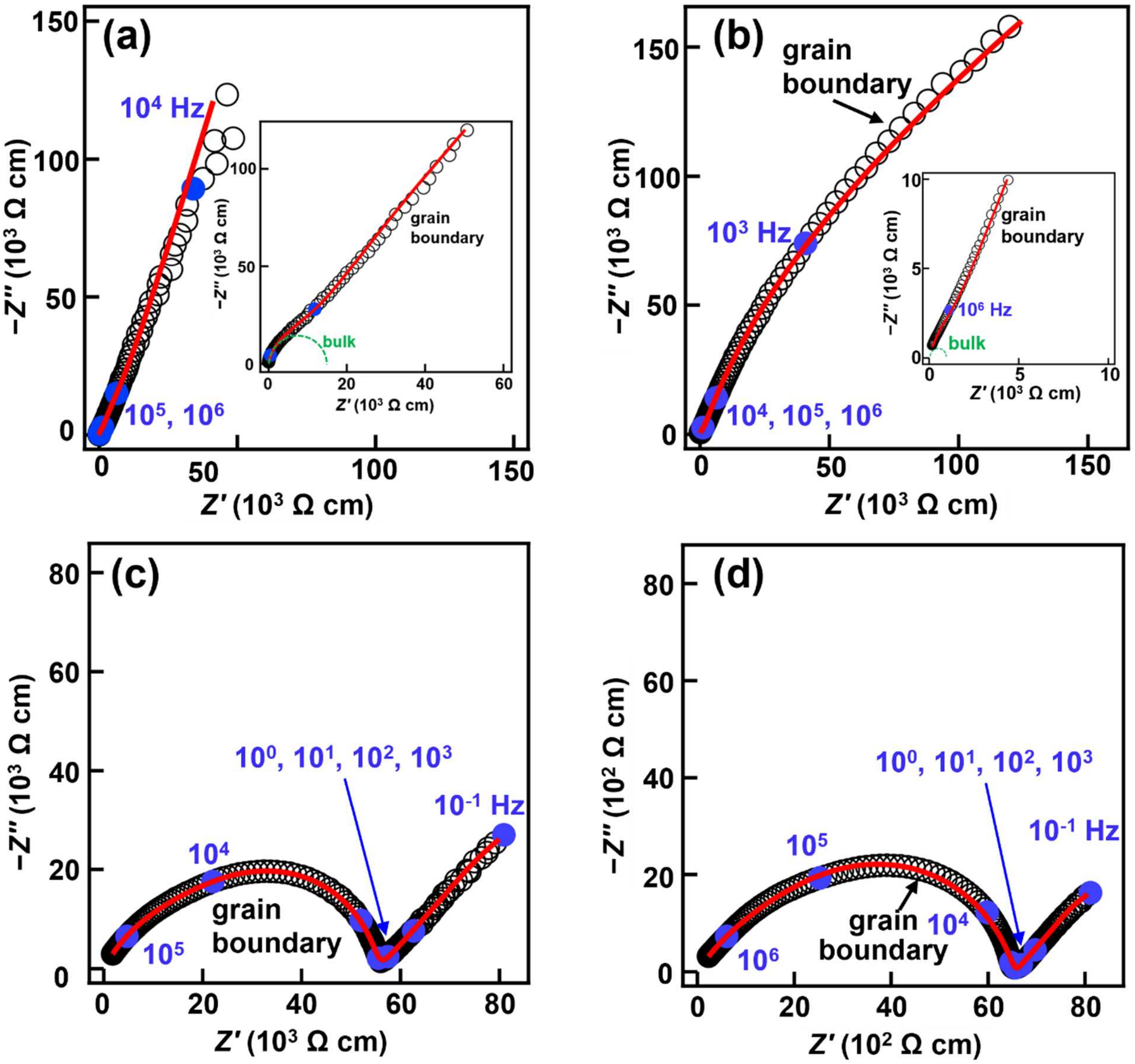

Figure S1. Complex impedance plots of BSM10W10 at (a) 49, (b) 122, (c) 204, and (d) 303 °C in wet air. Each number denotes the frequency at a blue closed circle. The red solid line represents the fitting curve.

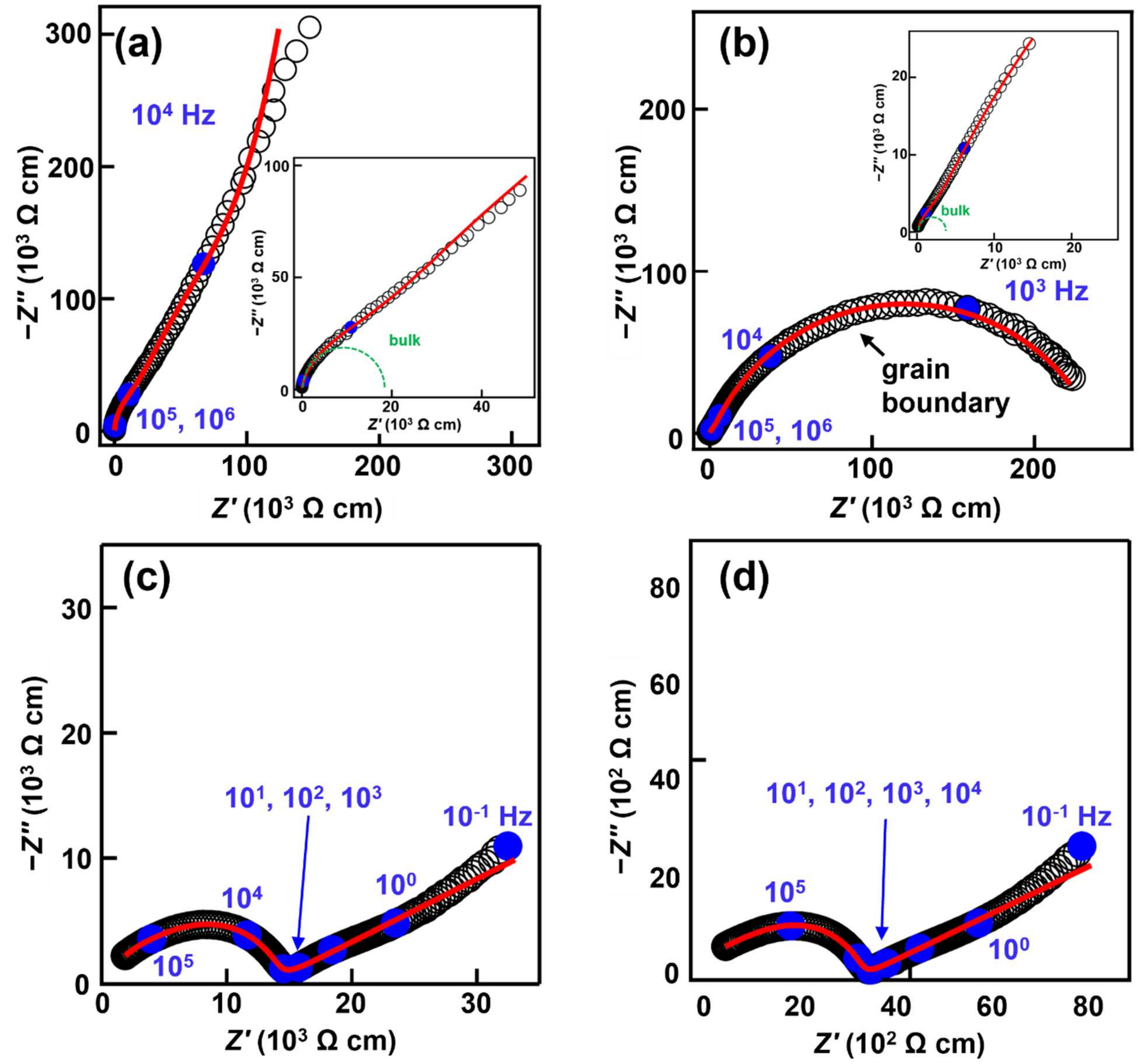

Figure S2. Complex impedance plots of BSM9W9 at (a) 48, (b) 123, (c) 206, and (d) 261 °C in wet air. Each number denotes the frequency at a blue closed circle. The red solid line represents the fitting curve.

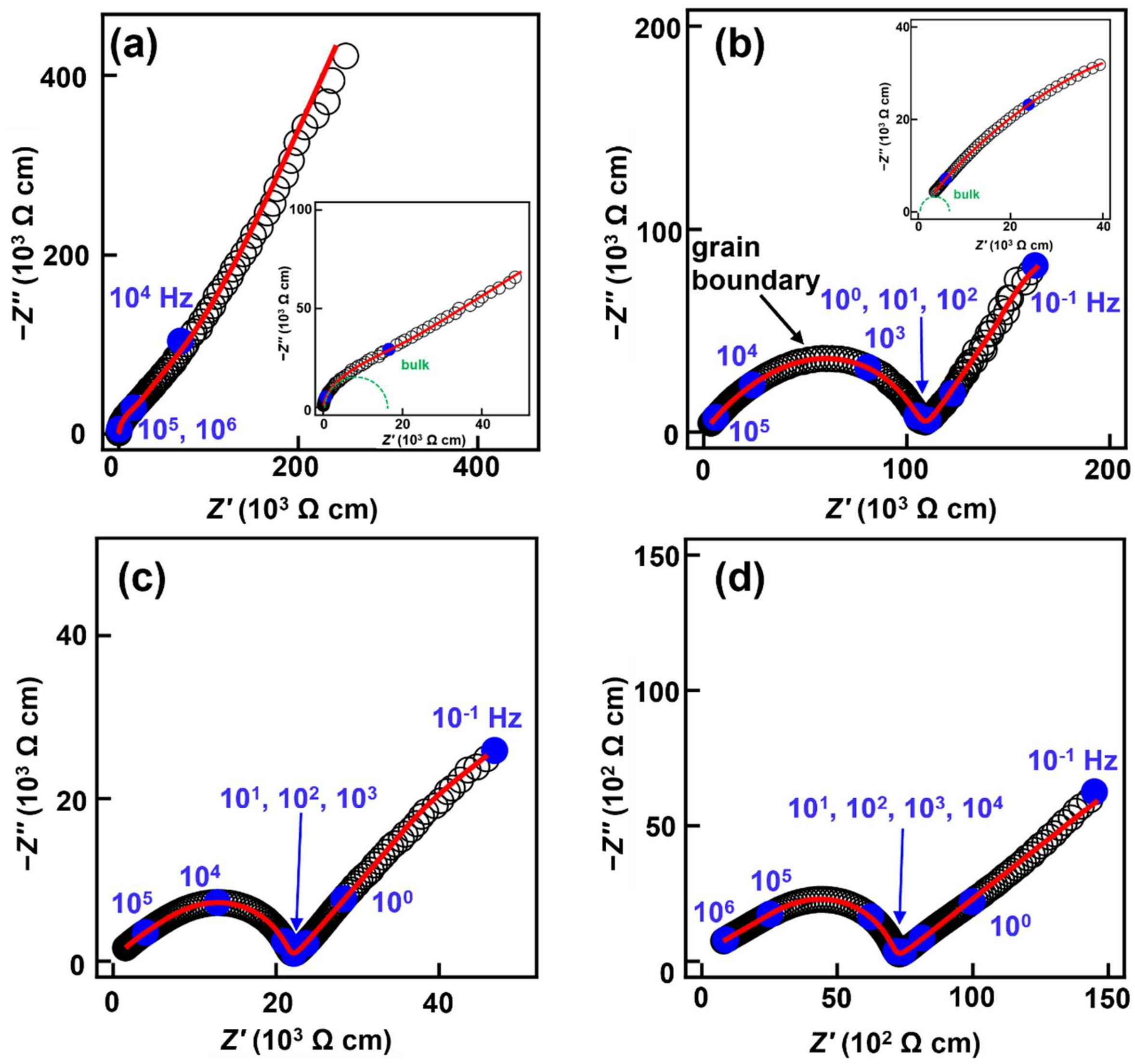

Figure S3. Complex impedance plots of BSM11W11 at (a) 49, (b) 147, (c) 204, and (d) 244 °C in wet air. Each number denotes the frequency at a blue closed circle. The red solid line represents the fitting curve.

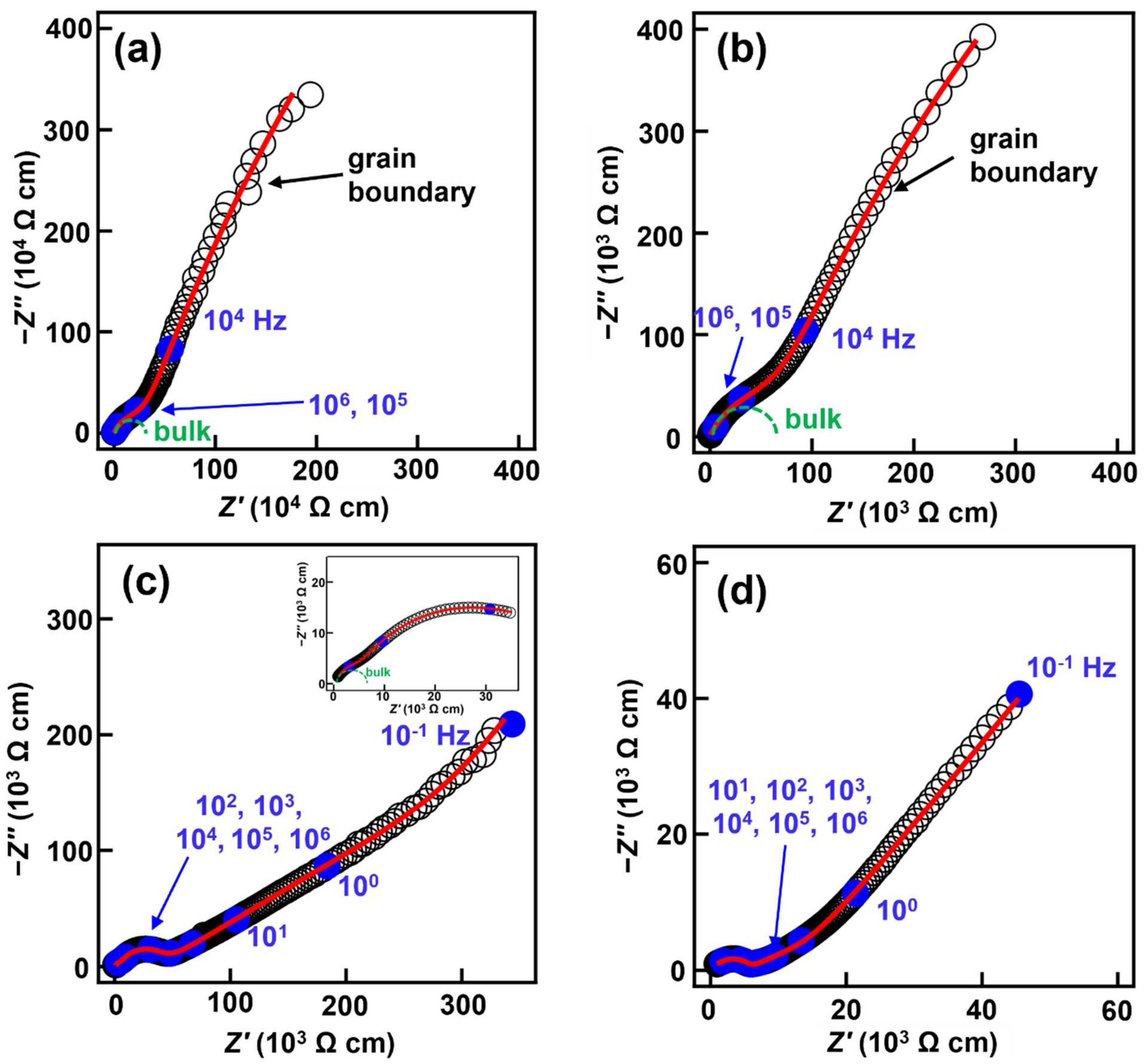

Figure S4. Complex impedance plots of BSW25 at (a) 45, (b) 88, (c) 140, and (d) 188 °C in wet air. Each number denotes the frequency at a blue closed circle. The red solid line represents the fitting curve.

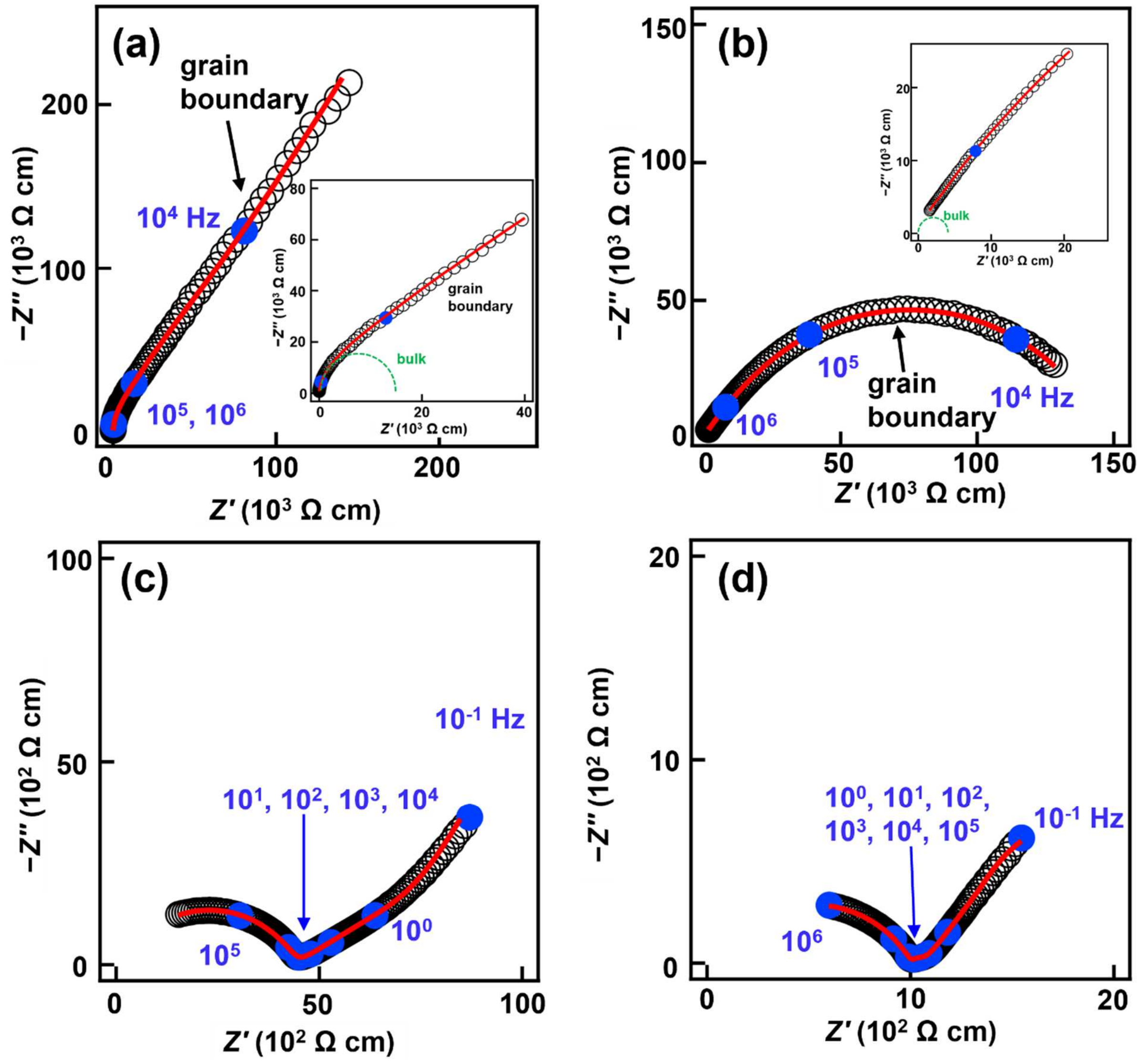

Figure S5. Complex impedance plots of BSM5W15 at (a) 45, (b) 111, (c) 218, and (d) 295 °C in wet air. Each number denotes the frequency at a blue closed circle. The red solid line represents the fitting curve.

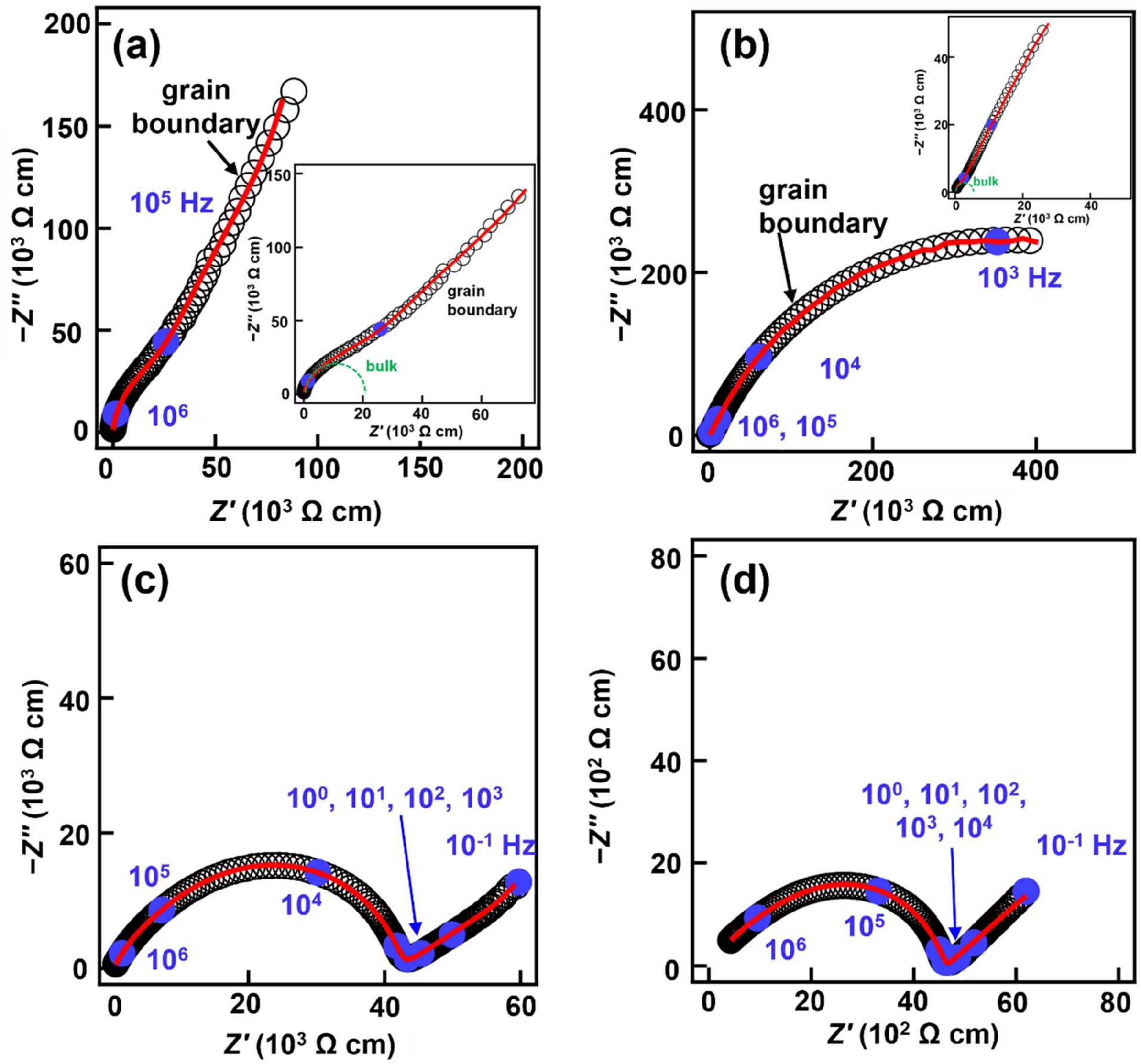

Figure S6. Complex impedance plots of BSM9W11 at (a) 49, (b) 121, (c) 204, and (d) 303 °C in wet air. Each number denotes the frequency at a blue closed circle. The red solid line represents the fitting curve.

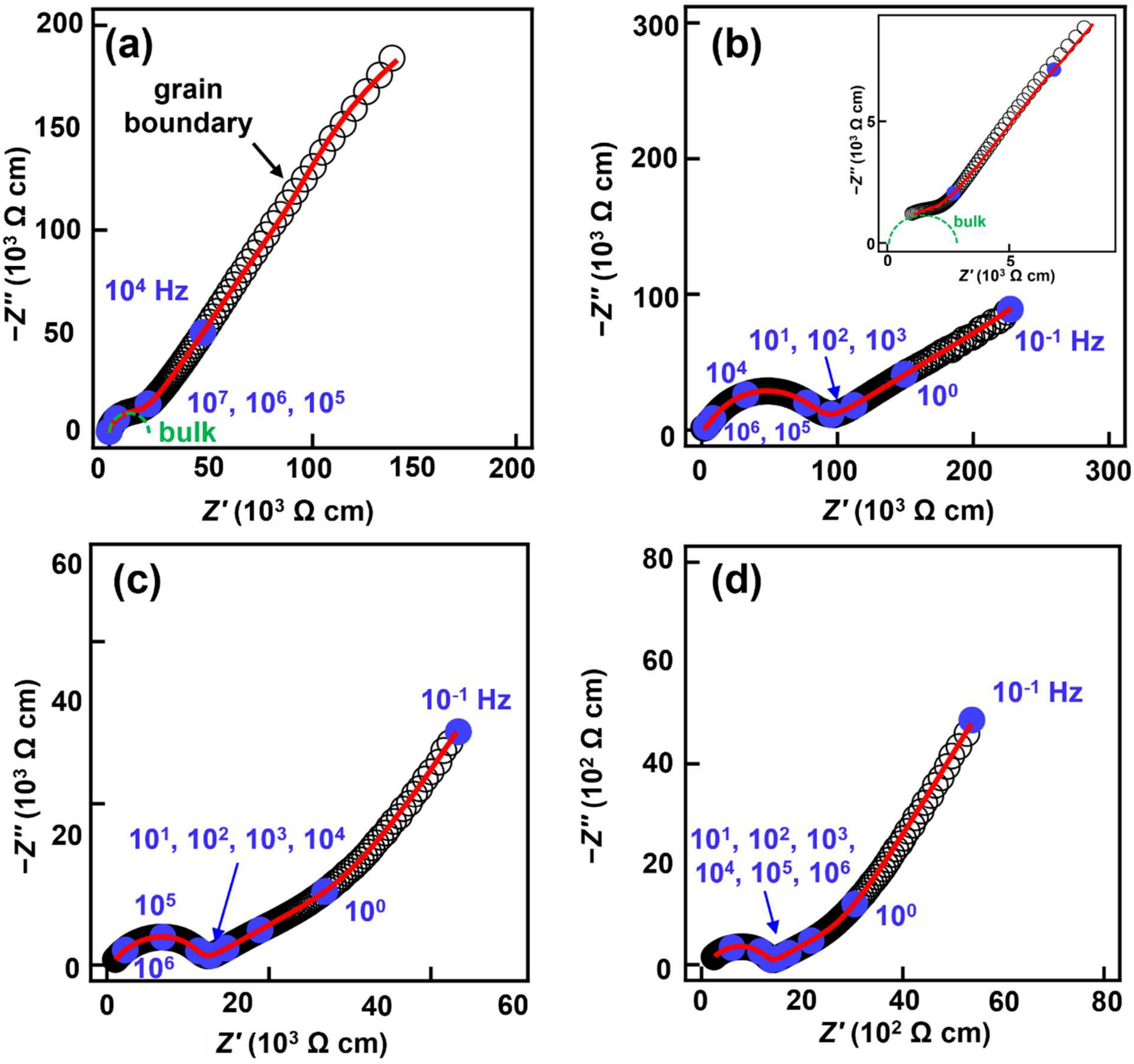

Figure S7. Complex impedance plots of BSM11W9 at (a) 49, (b) 121, (c) 204, and (d) 303 °C in wet air. Each number denotes the frequency at a blue closed circle. The red solid line represents the fitting curve.

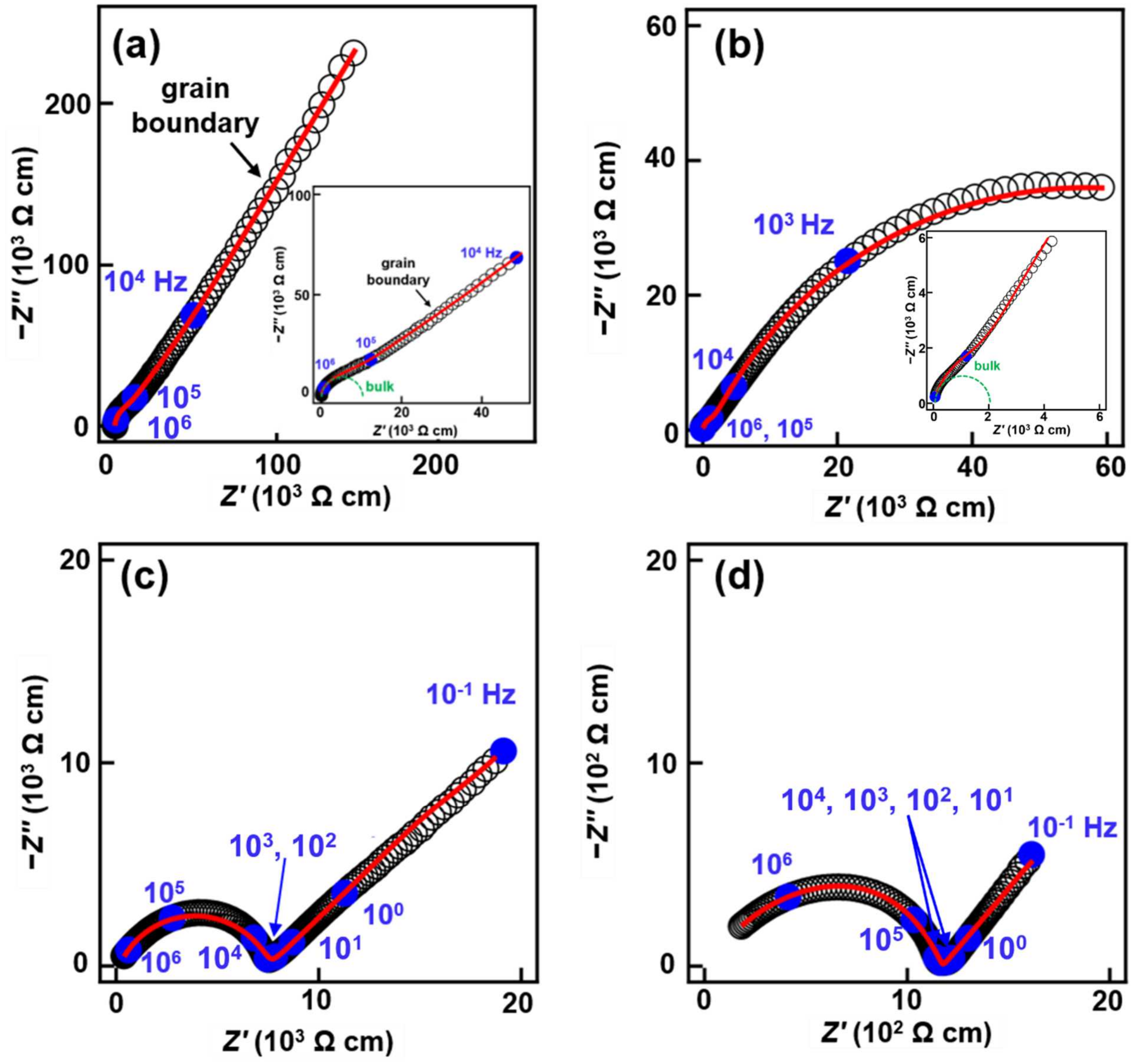

Figure S8. Complex impedance plots of BSM15W5 at (a) 46, (b) 114, (c) 194, and (d) 296 °C in wet air. Each number denotes the frequency at a blue closed circle. The red solid line represents the fitting curve.

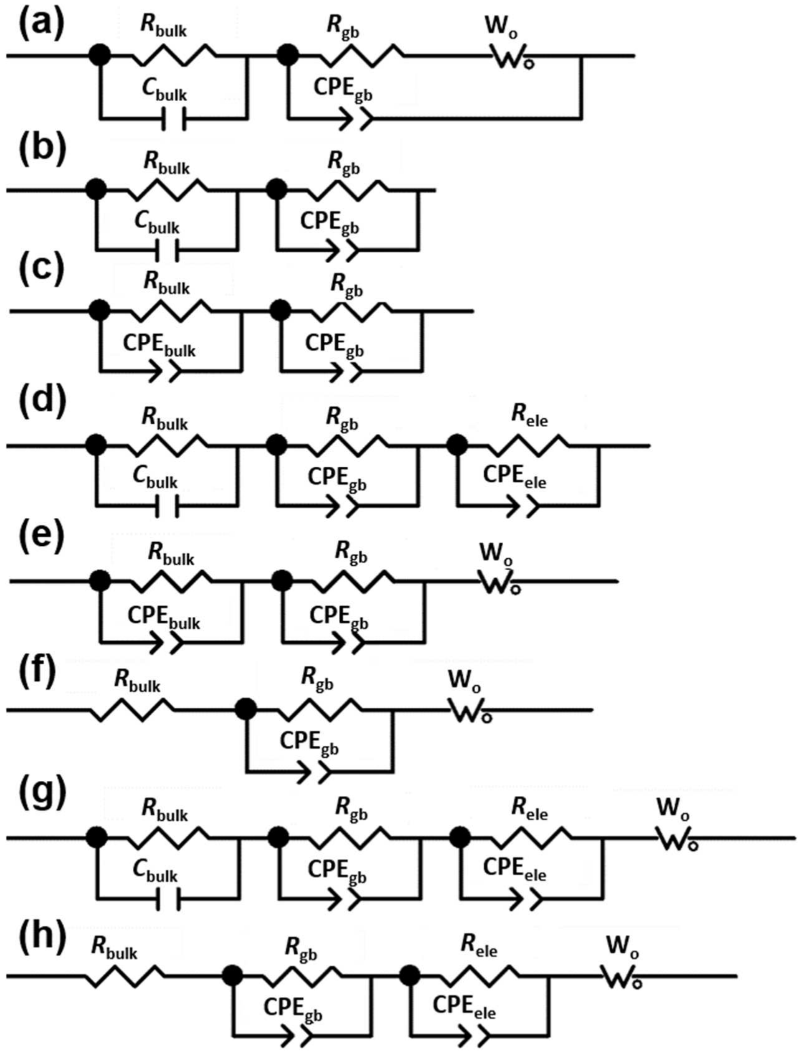

Figure S9. Equivalent circuits used to model the impedance spectra of BSM10W10 at (a) 49 °C, 72 °C, 95 °C, (d) 122 °C, 152 °C, and (h) 173–308 °C, BSM9W9 at (a) 44 °C, 72 °C, 96 °C, (c) 123 °C, (f) 152 °C, and (h) 174–261 °C, BSM11W11 at (a) 44 °C, 93 °C, and (f) 147–244 °C, BSW25 at (a) 45 °C, 88 °C, (c) 140 °C, 188 °C, and (e) 230–330 °C, BSM5W15 at (a) 45 °C, 65 °C, 87 °C, (d) 111 °C, (f) 140–246 °C, (h) 295 °C, BSM9W11 at (a) 45 °C, 65 °C, 86 °C, (b) 122 °C, and (g) 152–307 °C, BSM11W9 at (a) 46 °C, 67 °C, 89 °C, (g) 115 °C, 143 °C, (h) 163 °C, 194 °C, and (f) 220 °C–296 °C, BSM15W5 at (a) 46 °C, 67 °C, 89 °C, (b) 114 °C, (f) 194 °C, 211 °C, and (h) 247 °C, 296 °C. *R*, *C*, CPE, and W_o denote a resistance, capacitance, constant phase element, and open Warburg element, respectively. The subscripts “bulk”, “gb”, and “ele” denote the bulk, grain boundary, and electrode, respectively.

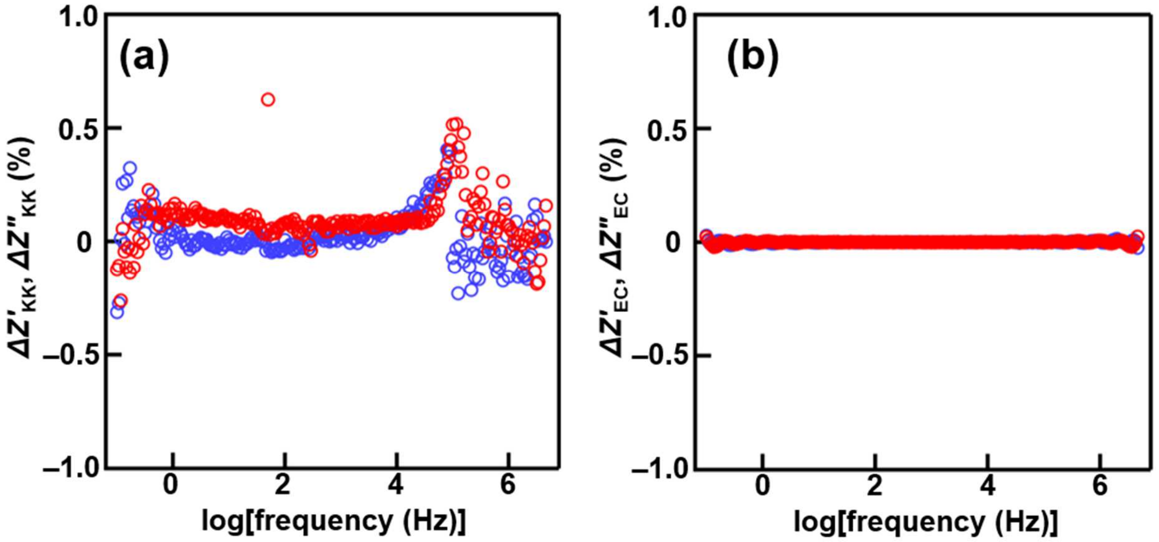

Figure S10. Residual plots of (a) the measured impedance data obtained by the Kramers-Kronig transformation and (b) the equivalent circuit fitting of BSM10W10 in wet air at 303 °C.[53-55]

Table. S2. Resistances for bulk R_b and grain boundary R_{gb} and capacitances for bulk C_b and grain boundary C_{gb} of BSM10W10 in wet air.

T (°C)	R_b (Ω cm)	C_b (F)	R_{gb} (Ω cm)	C_{gb} (F)
49	1.0×10^{4}	1.8×10^{-11}		
71	5.9×10^{3}	2.0×10^{-11}		
96	2.0×10^{3}	1.8×10^{-11}		
122	7.6×10^{2}	3.0×10^{-11}	9.7×10^{4}	8.2×10^{-11}
152	2.3×10^{2}		9.2×10^{4}	5.6×10^{-11}
173	1.7×10^{2}		6.9×10^{4}	6.0×10^{-11}
204	7.2×10		3.2×10^{4}	6.2×10^{-11}
232	4.2×10		1.6×10^{4}	6.2×10^{-11}
261	2.4×10		7.9×10^{3}	5.9×10^{-11}
307	1.2×10		2.9×10^{3}	5.9×10^{-11}

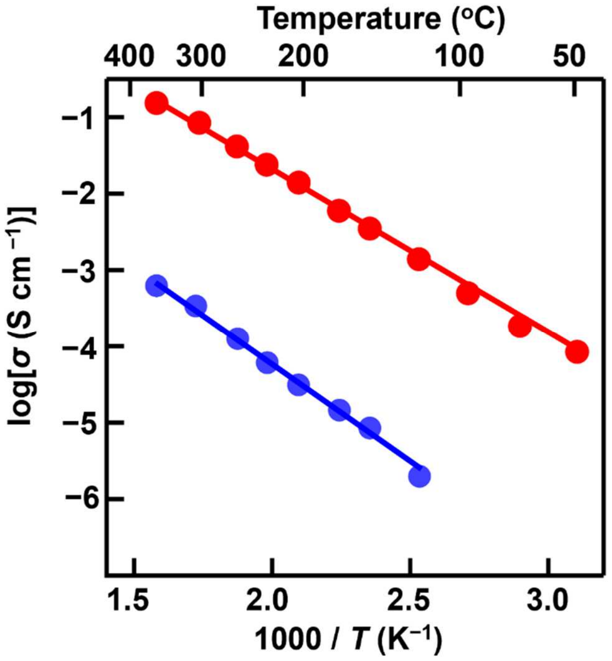

Figure S11. Arrhenius plots of bulk (red circles) and grain-boundary (blue circles) conductivities of BSM10W10 in wet air.

Table S3. H/D isotope effect of BSM10W10 on the activation energy E_a and pre-exponential factor *A*. Here, E_D and A_D are activation energy and pre-exponential factor of BSM10W10 in D_2O-saturated air, respectively. E_H and A_H are activation energy and preexponential factor of BSM10W10 in H_2O-saturated air, respectively.

Atmosphere	E_a (eV)	A (K S cm^{-1})	$E_D - E_H$ (eV)	A_H/A_D
H_2O-saturated air	0.42	1.3×10^5	0.04	0.46
D_2O-saturated air	0.46	2.8×10^5		

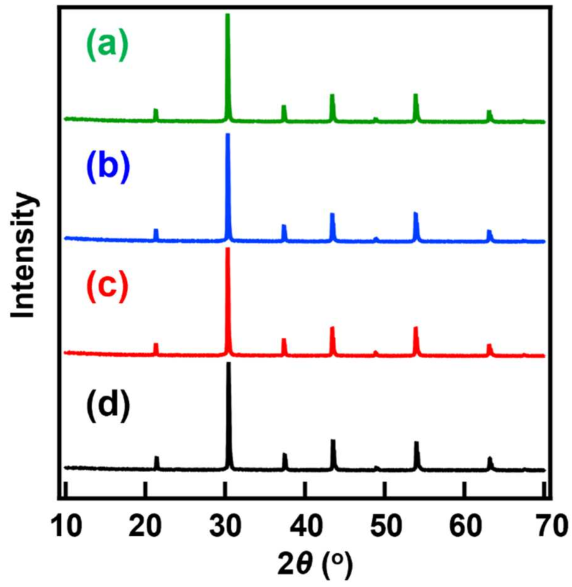

Figure S12. Cu Kα X-ray powder diffraction patterns of BSM10W10 after annealing at 250 °C for 24 h under (a) dry O_2, (b) dry H_2, and (c) dry CO_2, and (d) before annealing.

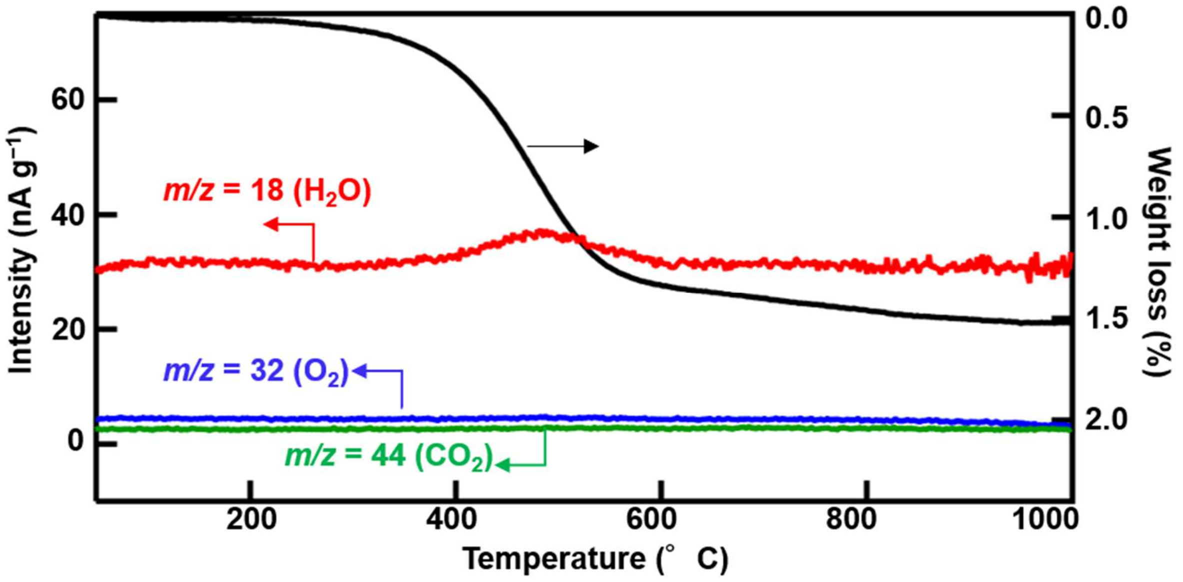

Figure S13. Thermogravimetric-mass spectrometric (TG-MS) data of wet powders of BSM10W10 measured under dry He flow. The sample weight decreased during heating. TG-MS data showed that the weight loss was due to the dehydration (water evaporation, *m/z* = 18, red line). CO_2 gas (*m/z* = 44, green line) and O_2 gas (*m/z* = 32, blue line) from the sample were not detected.

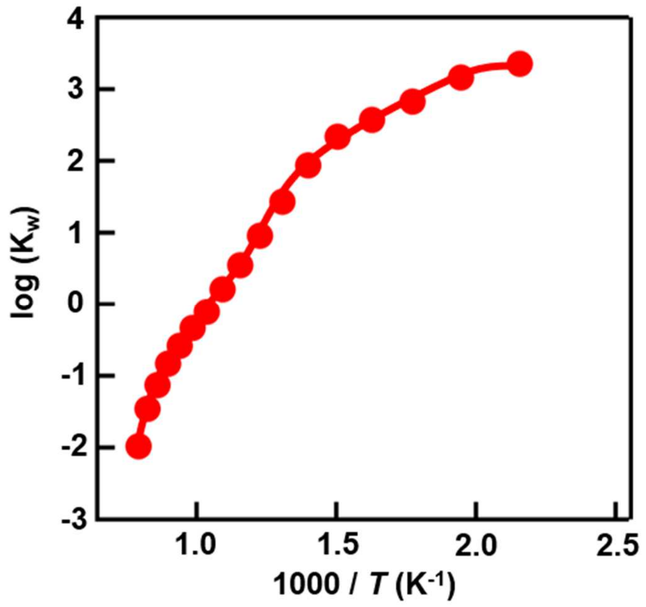

Figure S14. van 't Hoff plot of the equilibrium constant K_w for the hydration of BSM10W10. The K_w was calculated using the equation in the literature.[23]

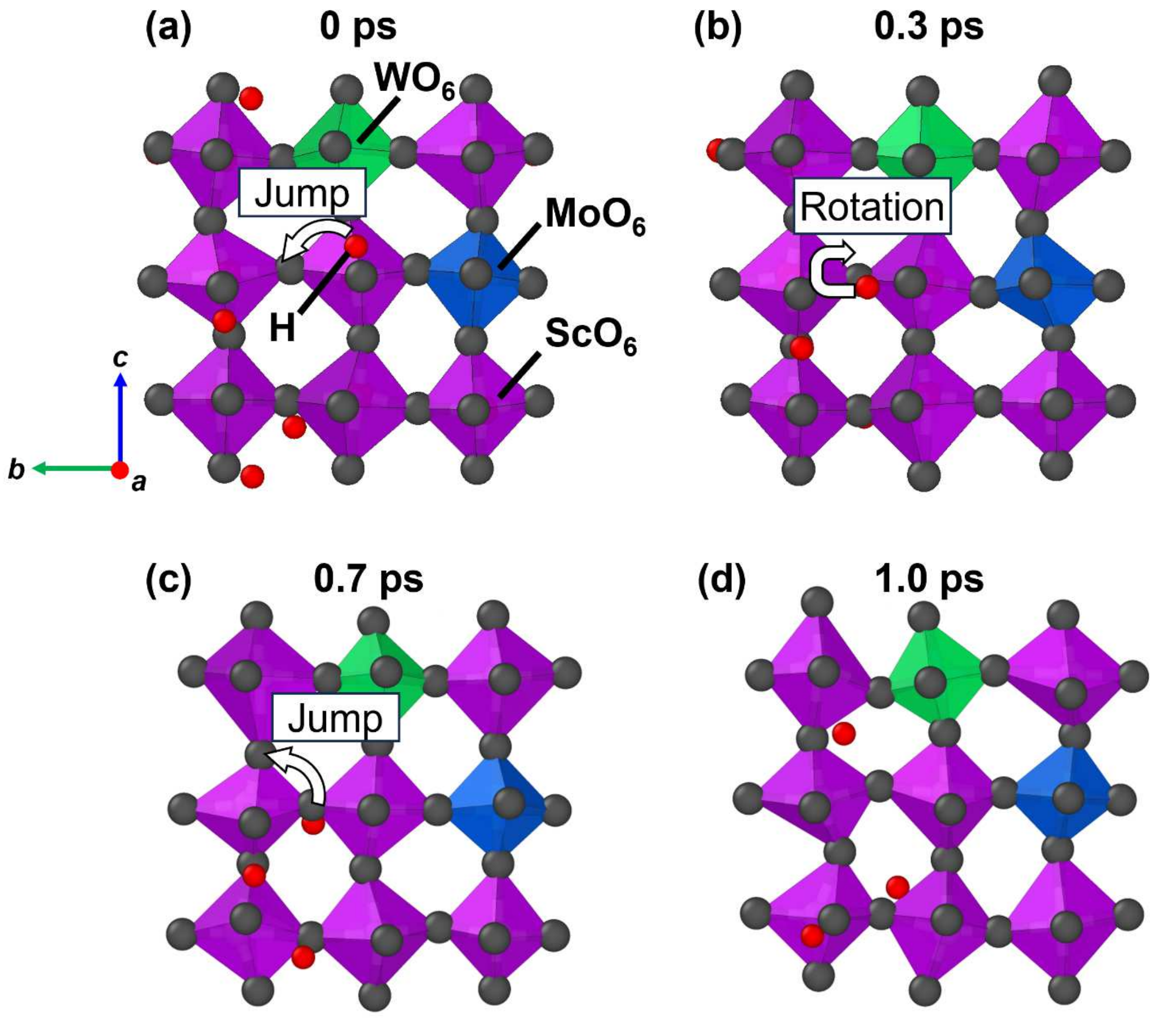

Figure S15. Snapshots of the atomic configuration in $Ba_{27}Sc_{23}M_2W_2O_{81}H_{15}$ showing the proton diffusion via Grotthuss mechanism from AIMD simulations (See also the movie).

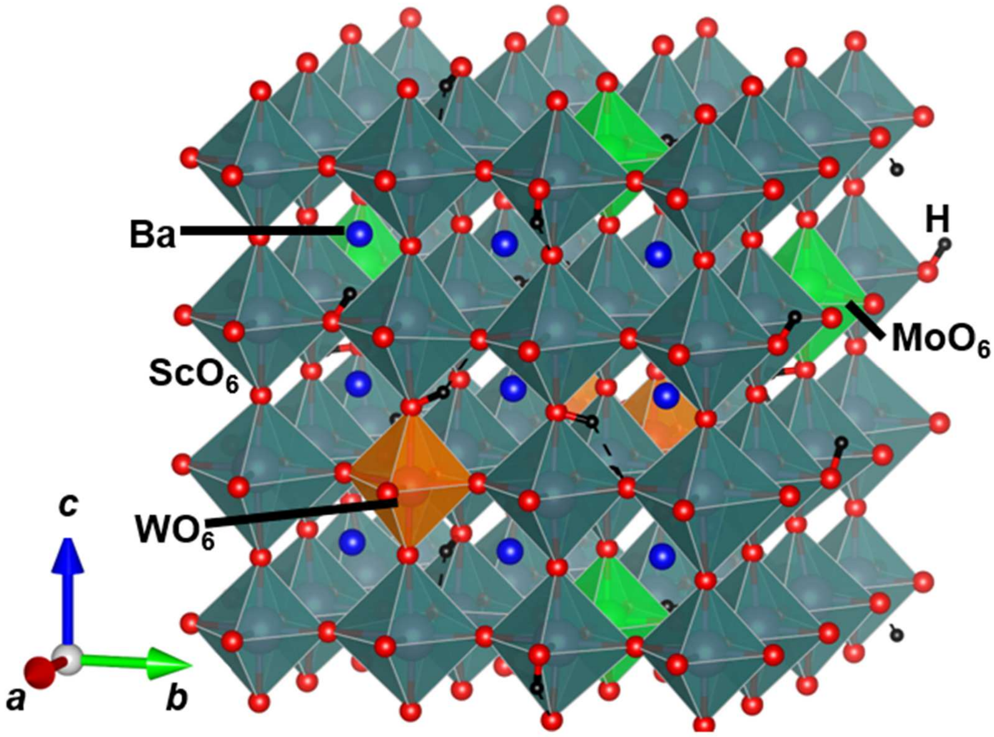

Figure S16. Optimized structure of $Ba_{27}Sc_{23}M_2W_2O_{81}H_{15}$, which was obtained by static DFT calculations.

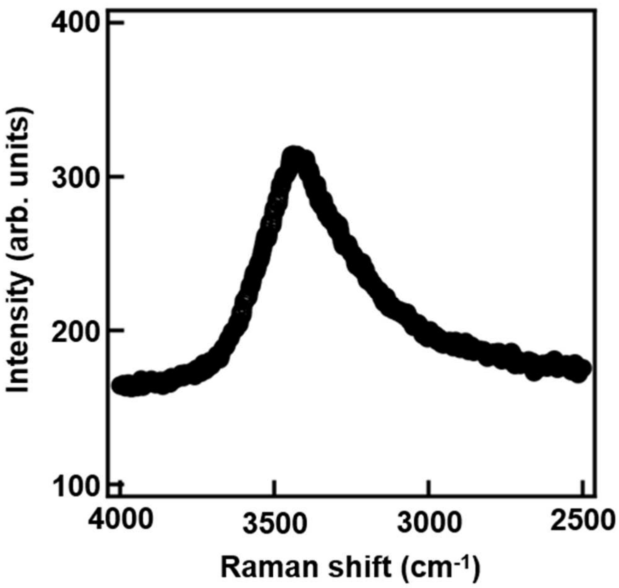

Figure S17. Raman spectra of wet powders of BSM10W10. Novak proposed an empirical equation to express the correlation between OH bond length and frequency using data of 21 materials[63]. Using the empirical equation, the OH bond length of the wet powders of BSM10W10 was estimated to be 0.99(6) Å from Raman data.

Table S4. Results of the refined crystal parameters and reliability factors in Rietveld analysis of the neutron diffraction data of $BaSc_{0.8}Mo_{0.1}W_{0.1}O_{2.6}(OD)_{0.4}$ (= $BaSc_{0.8}Mo_{0.1}W_{0.1}O_{2.8} \cdot 0.2\ D_2O$ = $BaSc_{0.8}Mo_{0.1}W_{0.1}O_{3.0}D_{0.4}$) at −243 °C.

Site, atom label *X*	Atom *Y*	$g(Y; X)^a$	Wyckoff position	x^a	y^a	z^a	$U_{iso}(Y; X)$ (Å^2)^b	BVS^c
Ba	Ba	1^d	1*b*	1/2	1/2	1/2	0.01519(4)	2.1
Sc/Mo/W	Sc	0.8^e	1*a*	0	0	0	0.01491(2)	3.4
	Mo	0.1^e	1*a*	= *x*(Sc)	= *y*(Sc)	= *z*(Sc)	= U_{iso}(Sc; Sc/Mo/W)	
	W	0.1^e	1*a*	= *x*(Sc)	= *y*(Sc)	= *z*(Sc)	= U_{iso}(Sc; Sc/Mo/W)	
O	O	1^e	3*d*	1/2	0	0	0.01415(2)	1.9
D	D	$1/60^f$	24*m*	0.4402(6)	0.2247(6)	0	0.0329(5)	1.0

Crystal system: cubic, Space group: $Pm\bar{3}m$, R_{wp} = 4.19%, R_B = 1.76%, R_F = 3.74%, Lattice parameter: a = 4.153984(3) Å.

^{a} Occupancy factor of *Y* atom at the *X* site. *x*, *y*, and *z*: atomic coordinates.

^{b} $U_{iso}(Y; X)$: Isotropic atomic displacement parameter of *Y* atom at the *X* site.

^{c} BVS: Bond valence sum. Here, the bond-valence parameters for Ba, Sc, Mo, W, and O atoms were referred from Ref.[64]. The bond-valence parameter of D atom was used for the calculation of BVS.[65]

^{d} Since the refined occupancy factors of Ba atom exceeded 1 in preliminary analysis, the occupancy factor of Ba atom was fixed to 1.

^{e} Since the obtained occupancy factors from the XRF results of Sc, Mo, and W atoms (Sc:Mo:W = 0.8:0.1:0.1), they were fixed to these values. The refined occupancy factor of O atom *g*(O; O) in a preliminary analysis was 1.0000(3). Thus, the occupancy factor of O atom was fixed to 1.

^{f} Since the refined occupancy factor of D atom (*g*(D) = 0.01667(7)) in a preliminary analysis agreed with that calculated from the TG data (*g*(D) = 1/60) within one estimated standard deviation, the occupancy factor of D atom was fixed to 1/60.

Supplementary Note

Here, we describe the details of the structural analyses. To investigate the proton concentration z and hydration of bulk BSM10W10, we performed Rietveld analyses of neutron diffraction data of the hydrated (deuterated) $BaSc_{0.8}Mo_{0.1}W_{0.1}O_{2.8-z/2}(OD)_z$ pellet (= $BaSc_{0.8}Mo_{0.1}W_{0.1}O_{2.8}\cdot(z/2)$ D_2O; BSM10W10) at −243 °C. In a preliminary analysis, the occupancy factor of O atom g(O; O) was refined to be 1.0000(3), which indicated the full hydration where the O site was fully occupied by O atoms. In another preliminary analysis, the proton (deuteron) concentration z calculated from the refined occupancy factor of proton (deuteron) (z = 0.4001(17)), which agreed with the value estimated from the TG measurements (z = 0.4). In the final refinement, the calculated intensities based on the cubic $Pm\bar{3}m$ perovskite-type structure were in good agreement with the observed ones (Fig. 4a), giving reasonably small reliability factors (R_{wp} = 4.19%, R_B = 1.76%, R_F = 3.74%; Table S4). The refined lattice parameter of BSM10W10 (4.153984(3) Å) agreed well with that optimized by the DFT calculations (Fig. S16). The bond valence sums (BVSs) for Ba atom (2.1) and O atom (1.9) agreed well with their formal charges 2. The BVS for D atom (1.0) agreed well with its formal charge 1. The calculated average BVS value of Sc/Mo/W cation (3.4) also agreed with the averaged oxidation number of the Sc, Mo, and W cations (3.6). These results indicate the validity of the refined crystal structure of $BaSc_{0.8}Mo_{0.1}W_{0.1}O_{3.0}D_{0.4}$ (BSM10W10). The refined crystal structure of BSM10W10 in Fig. 4b shows the cubic $Pm\bar{3}m$ perovskite-type structure consisting of $(Sc_{0.8}Mo_{0.1}W_{0.1})O_{6.0}D_{0.8}$ octahedra and Ba atoms. The occupancy factors of oxygen atom and proton (deuteron) indicated that the chemical formula of hydrated (deuterated) $BaSc_{0.8}Mo_{0.1}W_{0.1}O_{2.8}$ was $BaSc_{0.8}Mo_{0.1}W_{0.1}O_{3.0}D_{0.4}$ where water D_2O was fully incorporated as OD hydroxide ions in bulk $BaSc_{0.8}Mo_{0.1}W_{0.1}O_{2.8}$.

Table S5. Hydration enthalpy and entropy of $BaSc_{0.8}Mo_{0.1}W_{0.1}O_{2.8-z/2}(OH)_z$ (BSM10W10), $BaSc_{0.8}W_{0.2}O_{2.8-z/2}(OH)_z$ (BSW20)[40], and $BaSc_{0.8}Mo_{0.2}O_{2.8-z/2}(OH)_z$ (BSM20)[23] at 500−1000 °C. The hydration enthalpy and entropy of BSM10W10 were estimated using the van 't Hoff plots (Figure S14).

Composition	ΔH^o (kJ mol^{-1})	ΔS^o (J K^{-1} mol^{-1})
$BaSc_{0.8}Mo_{0.1}W_{0.1}O_{2.8-z/2}(OH)_z$	−116(5)	−123(6)
$BaSc_{0.8}Mo_{0.2}O_{2.8-z/2}(OH)_z$	−115(4)	−130(4)
$BaSc_{0.8}W_{0.2}O_{2.8-z/2}(OH)_z$	−111(5)	−117(5)

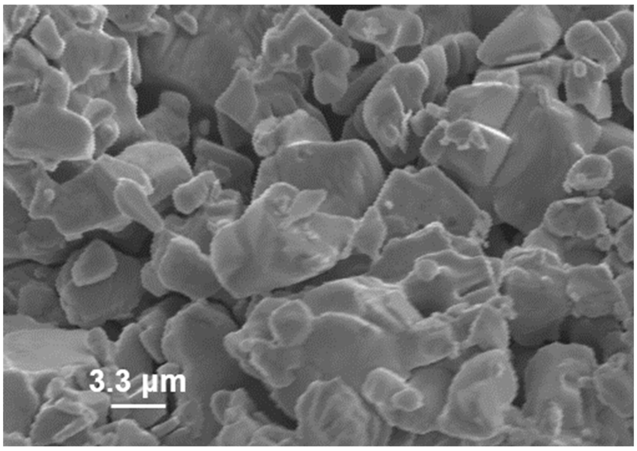

Figure S18. SEM micrograph of a sintered pellet of BSM10W10. The sample was thermally etched at 1600 °C for 1 hour prior to the SEM observation. The average grain size was estimated to be 4 μm in diameter.

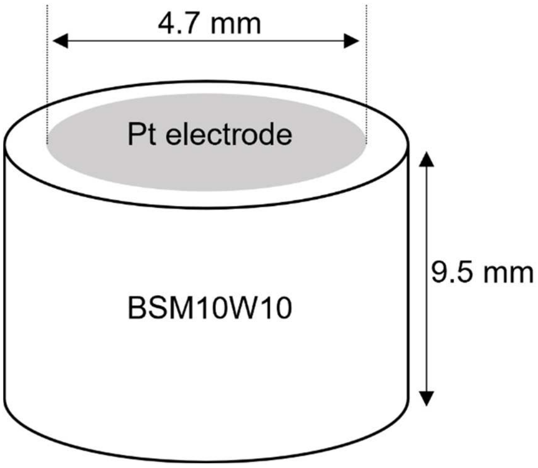

Figure S19. Specimen with dimensions for AC impedance measurements of BSM10W10.
